# Mechanical Properties and Leaching Characteristics of BF-MICP Solidified/Stabilized Ion-Type Rare Earth Tailings

**DOI:** 10.3390/microorganisms14081675

**Published:** 2026-07-30

**Authors:** Zhongqun Guo, Yukun Zhong, Jianqi Wu, Qiangqiang Liu, Xi Cao

**Affiliations:** 1 School of Civil Engineering, Jiangxi University of Science and Technology, Ganzhou 341000, China; 2 Jiangxi Provincial Key Laboratory of Water Ecological Conservation in Headwater Regions, Ganzhou 341000, China

**Keywords:** microbially induced calcium carbonate precipitation (MICP), basalt fiber (BF), biomineralization, ion-type rare earth tailings, solidification/stabilization

## Abstract

Ion-type rare earth tailings are mechanically weak and may release Pb and Zn, posing both geotechnical and environmental risks. Basalt-fiber-reinforced microbially induced carbonate precipitation (BF-MICP) was investigated as a combined solidification/stabilization treatment for these tailings. By integrating peak and post-peak mechanical responses, heavy-metal leaching, the spatial distribution of calcium carbonate (CaCO_3_), and microstructural characterization, this study distinguishes the respective contributions of microbial mineralization and fiber reinforcement. Tailings specimens were treated with basalt fiber contents ranging from 0 to 0.8% and evaluated using unconfined compression tests, leaching tests, CaCO_3_ measurements, X-ray diffraction, Fourier-transform infrared spectroscopy, and scanning electron microscopy with energy-dispersive spectroscopy. The unconfined compressive strength first increased and then decreased with increasing fiber content, reaching 1.72 MPa at 0.4% fiber, approximately 90% higher than that of the MICP-only group. At the same fiber content, compressive total energy absorption increased from approximately 18 to 68 kJ·m^−3^, indicating a marked improvement in post-peak toughness. BF-MICP treatment increased the CaCO_3_ content to approximately 2–3 times that of untreated tailings, although the deposits remained more abundant in the outer region than in the core, and the total CaCO_3_ content varied little with fiber dosage. The leached concentrations of Pb and Zn decreased by 79–81% and 81–84%, respectively, with no clear additional reduction as the fiber dosage increased. Microstructural analyses showed that calcite-dominated deposits connected tailing particles and fiber surfaces. These results indicate that MICP primarily governed mineral cementation and heavy-metal immobilization, whereas basalt fibers mainly improved load transfer, crack bridging, and post-peak structural integrity. A fiber content of 0.3–0.4% provided the best overall balance between mechanical performance and leaching control.

## 1. Introduction

Rare earth elements are widely used in new energy technologies, advanced materials, and electronic information industries. Ion-type rare earth ores are enriched in medium and heavy rare earth elements and thus constitute strategically important mineral resources [1,2]. In ion-type rare earth (weathered crust elution-deposited) deposits, rare earth ions are predominantly adsorbed on the surfaces of clay minerals in an exchangeable form, making it difficult to directly concentrate them using conventional mining and beneficiation processes [3], thereby prompting the successive adoption of vat leaching, heap leaching, and in-situ leaching for industrial production and resource development [4,5,6,7]. Prolonged leaching triggers a series of physicochemical reactions within the ore body, weakening the skeletal framework, inducing localized sandification, and causing strength deterioration [8]. Meanwhile, heavy metals such as Pb and Zn ions can be released, mobilized, and accumulated at soil–water interfaces, thereby exacerbating the instability/failure of rare earth tailings and associated environmental risks [9,10]. The generation of large quantities of rare earth tailings not only occupies valuable land resources but also poses a serious threat to ecological security in mining areas and surrounding regions. Meanwhile, the demand for construction fill materials in geotechnical and foundation engineering is substantial, and the resulting resource consumption and environmental pressures are increasingly evident. Therefore, developing cost-effective approaches for treating rare earth tailings has become a key solution for the resource utilization of mining solid wastes. In particular, valorizing rare earth tailings for applications such as foundation filling and related geotechnical works is a critical pathway.

Currently, solidification/stabilization has increasingly emerged as a promising engineering treatment technology in recent years because it can simultaneously strengthen tailings structure and stabilize heavy-metal speciation through the combined effects of physical encapsulation and chemical cementation [11,12,13]. To reduce the potential environmental risks of tailings and alleviate safety hazards such as ore-body erosion and instability, many researchers have explored feasible strategies for the valorization of solid wastes. Microbially induced carbonate precipitation (MICP) is increasingly regarded as a promising biotreatment technology owing to its eco-friendly nature, relatively low energy demand, and potential to concurrently achieve soil improvement and heavy-metal stabilization within a single system [14,15]. This technique relies on ureolytic bacteria to hydrolyze urea and, in the presence of Ca^2+^, generate CaCO_3_ cementation phases that strengthen the soil; meanwhile, it reduces heavy-metal mobility through adsorption, encapsulation, and co-precipitation processes [16,17]. However, the CaCO_3_ cementation phases produced by MICP remain brittle, resulting in limited post-peak load-bearing capacity and rapid crack propagation, and thus insufficient ductility and energy dissipation of the treated material [18]. To address this limitation, a “fiber reinforcement-biocementation” composite approach has been proposed, in which short fibers are introduced into the biomineralization system to form a three-dimensional load-bearing framework [19,20]. Basalt fiber, a natural inorganic silicate fiber, features high tensile strength, a high elastic modulus, and good chemical stability and durability, and can be used to mitigate the brittleness and insufficient post-peak load-bearing capacity of CaCO_3_-cemented matrices [21]. Zhang et al. [22] reported in a study on crack repair of marine mortar that basalt fiber can adsorb Ca^2+^ and promote CaCO_3_ growth on the fiber surface, thereby enhancing fiber bridging and densifying the mineralized products. Mehmood et al. [23] combined basalt fiber with enzyme-induced carbonate precipitation (EICP) for expansive-soil improvement and indicated that carbonate deposition and fiber bridging/interfacial enhancement exert synergistic reinforcement effects. Despite the increasing interest in fiber-reinforced biocementation, the application of basalt-fiber-reinforced MICP to ion-type rare earth tailings remains insufficiently understood. Existing studies have largely focused on strength improvement, whereas post-peak behavior, energy dissipation, the spatial distribution of mineralization products, and the relationship between mechanical reinforcement and heavy-metal immobilization have received limited attention. In particular, it remains unclear whether basalt fibers directly contribute to Pb and Zn immobilization or primarily improve the mechanical integrity of the MICP-treated matrix. Clarifying these issues is important for evaluating the engineering applicability and environmental safety of BF-MICP-treated tailings.

Accordingly, this study investigated the solidification/stabilization of ion-type rare earth tailings using MICP combined with basalt fibers at dosages of 0–0.8%. The effects of fiber dosage on UCS, residual stress, energy dissipation, CaCO_3_ content and spatial distribution, Pb and Zn leaching, heavy-metal speciation, and microstructural characteristics were systematically evaluated to determine the optimal fiber dosage and clarify the underlying mechanisms. The results showed that a fiber dosage of 0.3–0.4% provided the best overall mechanical performance. MICP primarily governed CaCO_3_ cementation and Pb/Zn immobilization, whereas basalt fibers mainly improved load transfer, crack bridging, and post-peak structural integrity. These findings provide a basis for the engineering reuse of ion-type rare earth tailings and clarify the different functions of microbial mineralization and fiber reinforcement.

## 2. Materials and Methods

### 2.1. Experimental Materials

#### 2.1.1. Ion-Type Rare Earth Tailings

**Figure 1:**
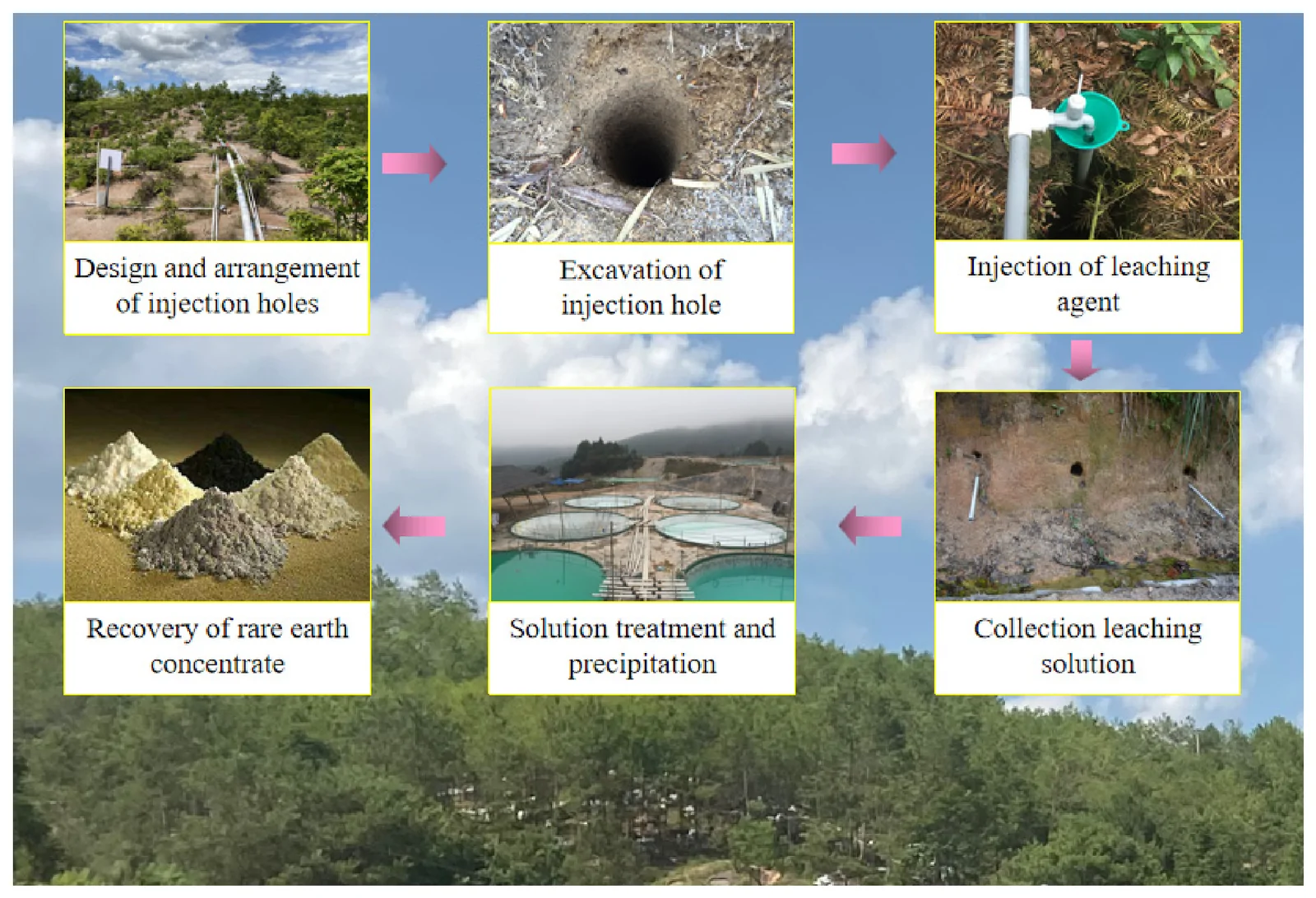
Main process of in-situ leaching.

The rare earth tailings used in this study were collected from an ion-type rare earth mining area in Fujian Province, China. The production of ion-type rare earth ores mainly involves ore-body exploration and preparation, design of injection wells and boreholes, solution injection and leaching, collection of rare earth pregnant liquor, solution treatment, and rare earth precipitation. The in-situ leaching process is illustrated in Figure 1.

Field surveys showed visible traces of leaching-agent runoff along the slope surfaces, accompanied by extensive exposure of the tailings cover layer. Under natural conditions, the heavy-metal contents of tailings soil in this area exhibited substantial spatial heterogeneity, with Pb of 55–520 mg/kg and Zn of 81–300 mg/kg, indicating potential mobility risks [24]. To reduce uncertainties arising from spatial variability in sampling, in-situ sampling was performed in an area not disturbed by leaching activities, and the collected samples were used for laboratory-scale simulated leaching. To simulate the leaching process of ion-type rare earth ores, an MgSO_4_ solution—commonly used as a leaching agent in the mine—was employed for laboratory leaching: soil samples were immersed in an MgSO_4_ solution with pH 2 and a mass fraction of 6% and left to stand for 7 d. After leaching, the samples were subjected to subsequent BF-MICP treatment and related tests [25]. The Pb^2+^ and Zn^2+^ contents in the rare earth tailings were both controlled at 500 mg/kg.

**Figure 2:**
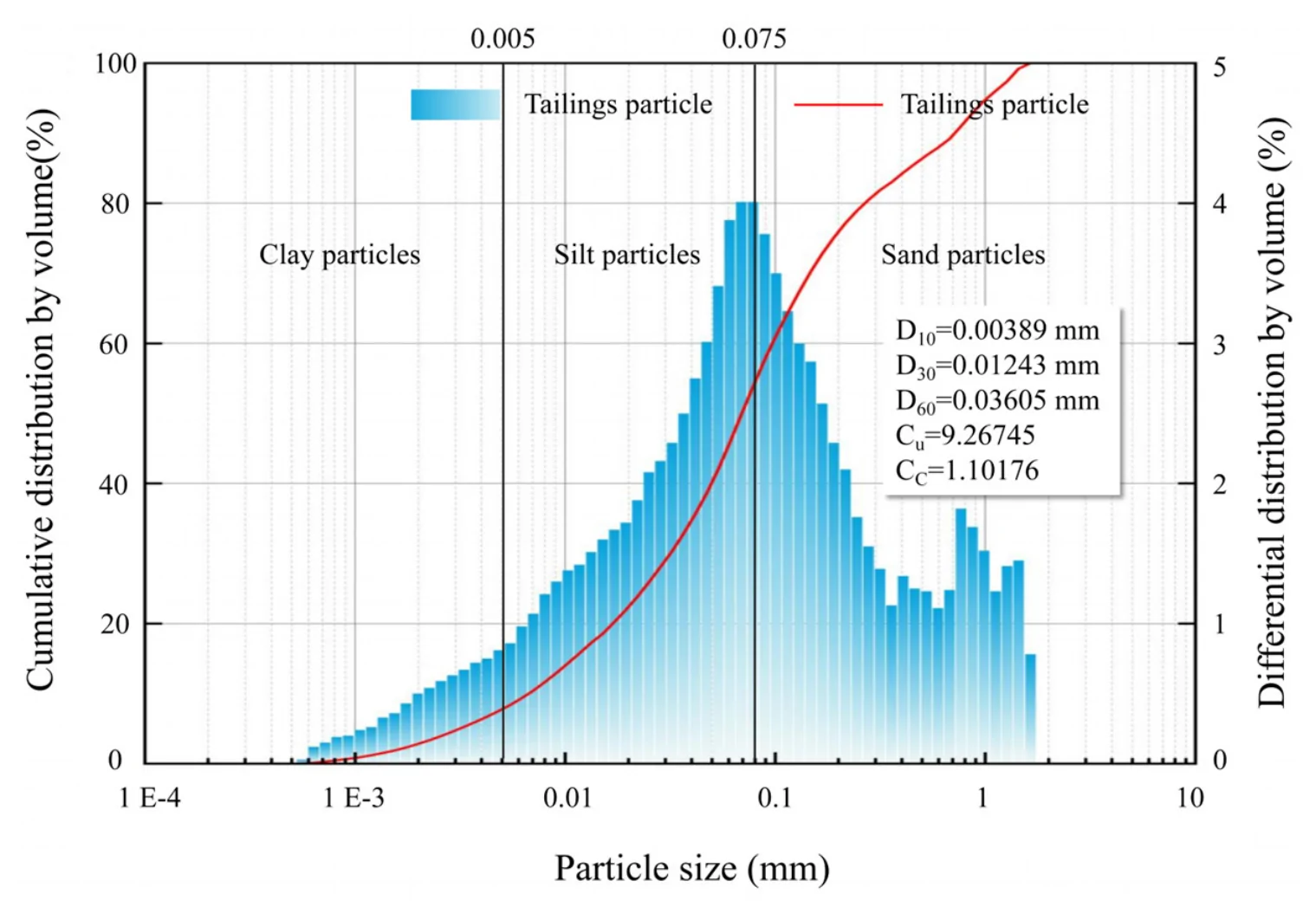
Particle-size distribution of ion-type rare earth tailings.

Basic physical property tests were performed to determine the natural water content, density, liquid limit, plastic limit, and pH of the tailings soil, as summarized in Table 1. The particle-size distribution curve of the tailings soil is shown in Figure 2. Based on the indices in Table 1 and Figure 2, the tailings soil was classified as low-plasticity silty clay.

The characteristic particle sizes were $D_{10}=0.00389\mathrm{mm}$, $D_{30}=0.01243\mathrm{mm}$, and $D_{60}=0.03605\mathrm{mm}$, with Cu=9.27andCc=1.10. A first-order estimate based on the Kovacs relationship indicated that the controlling pore-throat diameter was on the order of 1–3 μm; however, this value should be regarded as an approximate estimate because of the fine-grained and heterogeneous nature of the tailings.

**Figure 3:**
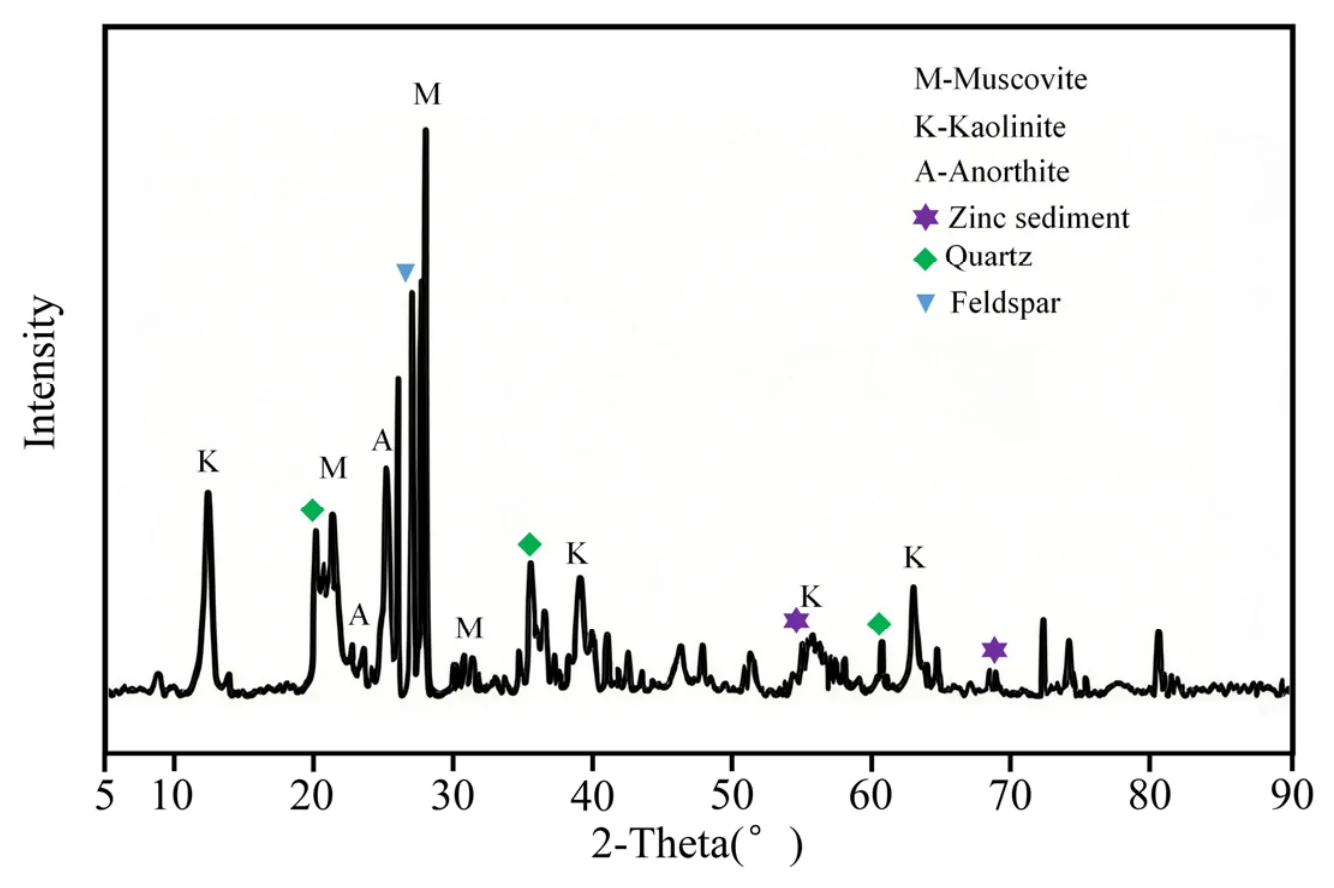
XRD patterns of rare earth tailings after laboratory-simulated leaching.

The bulk chemical composition of the tailings was determined by X-ray fluorescence (XRF), and the major oxide contents are listed in Table 2. The mineralogical composition was characterized by X-ray diffraction (XRD), with a representative diffractogram shown in Figure 3. The XRD results indicate that the tailings mainly consist of muscovite, kaolinite, quartz, and feldspar.

#### 2.1.2. Chopped Basalt Fiber

Chopped basalt fiber (BF) was selected as the reinforcing material in this study. The good chemical stability and durability of basalt fiber enable it to withstand acidic leaching and frequent wet–dry (rainy-season) cycles at ion-type rare earth tailings sites [26,27], which helps maintain a stable fiber–soil interface and mitigate strength deterioration during biomineralization. The fiber was produced from natural basalt by high-temperature melting and drawing through a platinum bushing, yielding a high-modulus inorganic mineral fiber. The physical properties of the basalt fiber are provided in Table 3. For example, a fiber content of 0.4% indicates that 0.4 g of basalt fiber was added to every 100 g of dry tailings.

**Figure 4:**
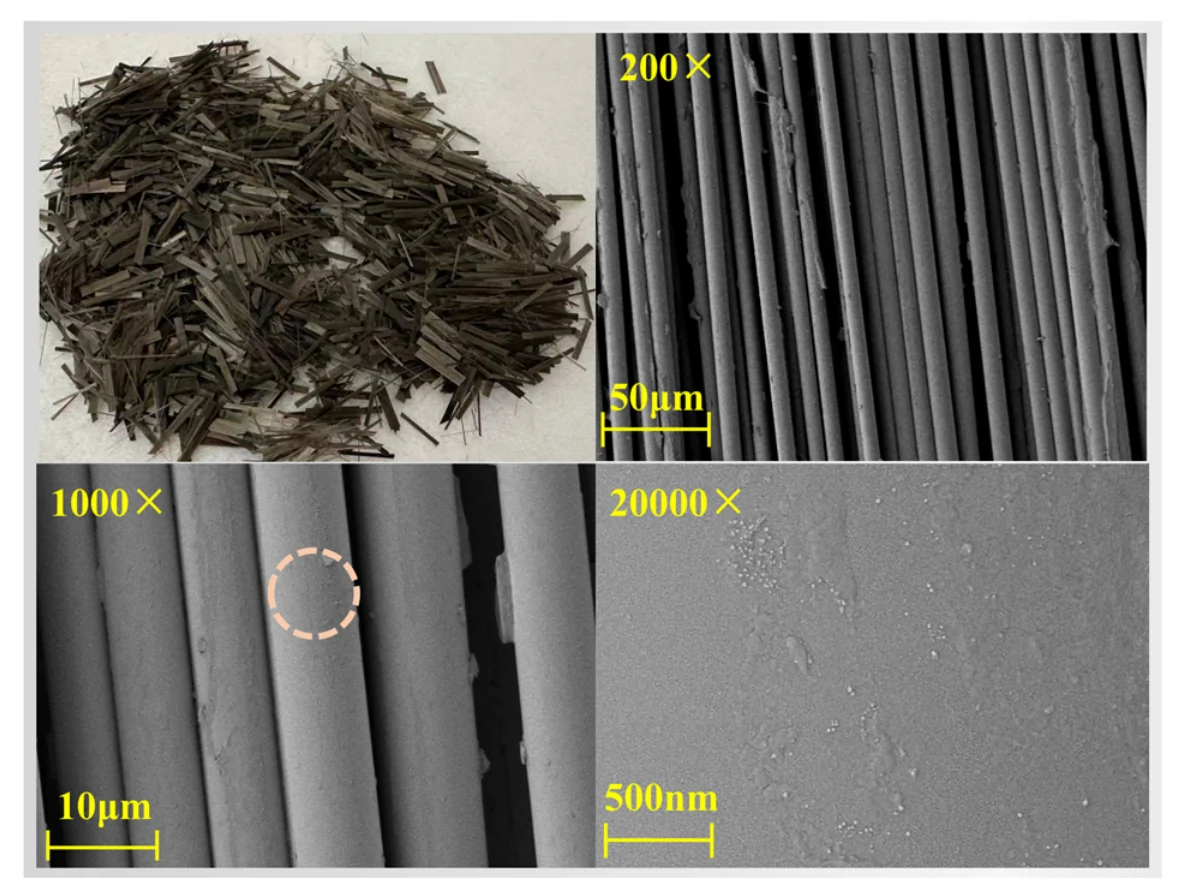
Macroscopic morphology and microstructure of basalt fibers.

The macro- and micro-morphologies of the basalt fiber are shown in Figure 4. The fibers were in a chopped form, and their surfaces were generally smooth, but exhibited scratches, micro-pits, and a small amount of particle attachment. These features are beneficial for the heterogeneous nucleation and deposition of CaCO_3_ during MICP, facilitating CaCO_3_ formation on the fiber surface and its connection with surrounding particles, thereby enhancing interfacial bonding and resistance to slippage between the fibers and tailings particles. To reduce fiber agglomeration and improve dispersion, a dry-mixing pre-dispersion procedure was adopted: the fibers were first uniformly mixed with the tailings soil by mass and then added in stages, with the fiber dosage controlled at 0.0–0.8% to ensure a relatively uniform spatial distribution and improve experimental reproducibility.

#### 2.1.3. Sporosarcina Pasteurii and Cementation Solution

The urease-producing bacterium Sporosarcina pasteurii (CGMCC 1.3687) selected in this study can secrete abundant urease during metabolism, catalyze urea hydrolysis to generate CO_3_^2−^ and induce CaCO_3_ precipitation in the presence of added Ca^2+^; therefore, it has been widely used in studies on biocementation of soils and solidification of heavy metals [28].

The strain was obtained from the China General Microbiological Culture Collection Center (CGMCC) and was revived and purified following standard procedures prior to inoculation. The culture medium contained (g·L^−1^) peptone 5.0, beef extract 3.0, and urea 20.0; for the solid medium, agar 20.0 (g·L^−1^) was additionally added, with deionized water as the solvent. After dissolution, the pH was adjusted to 7.0 ± 0.1 using 1 mol·L^−1^ NaOH, and the medium was autoclaved at 121 °C for 30 min. After pre-incubation at 30 °C and sterility testing, a single colony was inoculated into the same liquid medium and cultured under shaking conditions. After cultivation, the bacterial suspension collected at approximately 26 h was used as the grouting bacterial solution [29].

The cementation (grouting) solution was based on a urea-calcium chloride system, and the formulation is summarized in Table 4. Each 1 L of cementation solution contained CO(NH_2_)_2_ (60.0 g), CaCl_2_ (113.0 g), NH_4_Cl (10.0 g), NaHCO_3_ (2.0 g), and Nutrient broth (3.0 g); the solution was made up to 1 L with deionized water, and the initial pH was adjusted to 7.2. NH_4_Cl served as a nitrogen source for bacterial growth, whereas NaHCO_3_ acted as a buffering agent. Their combined use helped maintain a suitable pH under the acidic leaching conditions commonly encountered in ion-type rare earth tailings, thereby ensuring urease activity and biomineralization rate [30].

The efficiency of MICP is sensitive to temperature, pH, nutrient availability, treatment duration, and metal-ion concentration because these parameters regulate bacterial growth, urease activity, and carbonate precipitation. Unsuitable temperature or pH and excessive metal-ion concentrations may suppress microbial metabolism, whereas overly rapid precipitation at high reactant concentrations may promote near-surface clogging and restrict inward transport. A longer treatment period generally allows further carbonate accumulation until transport limitations become dominant. In the present study, these parameters were maintained constant to isolate the effect of basalt fiber dosage.

### 2.2. Experimental Methods

#### 2.2.1. Bacterial Cultivation and Microbial Activity Determination

**Figure 5:**
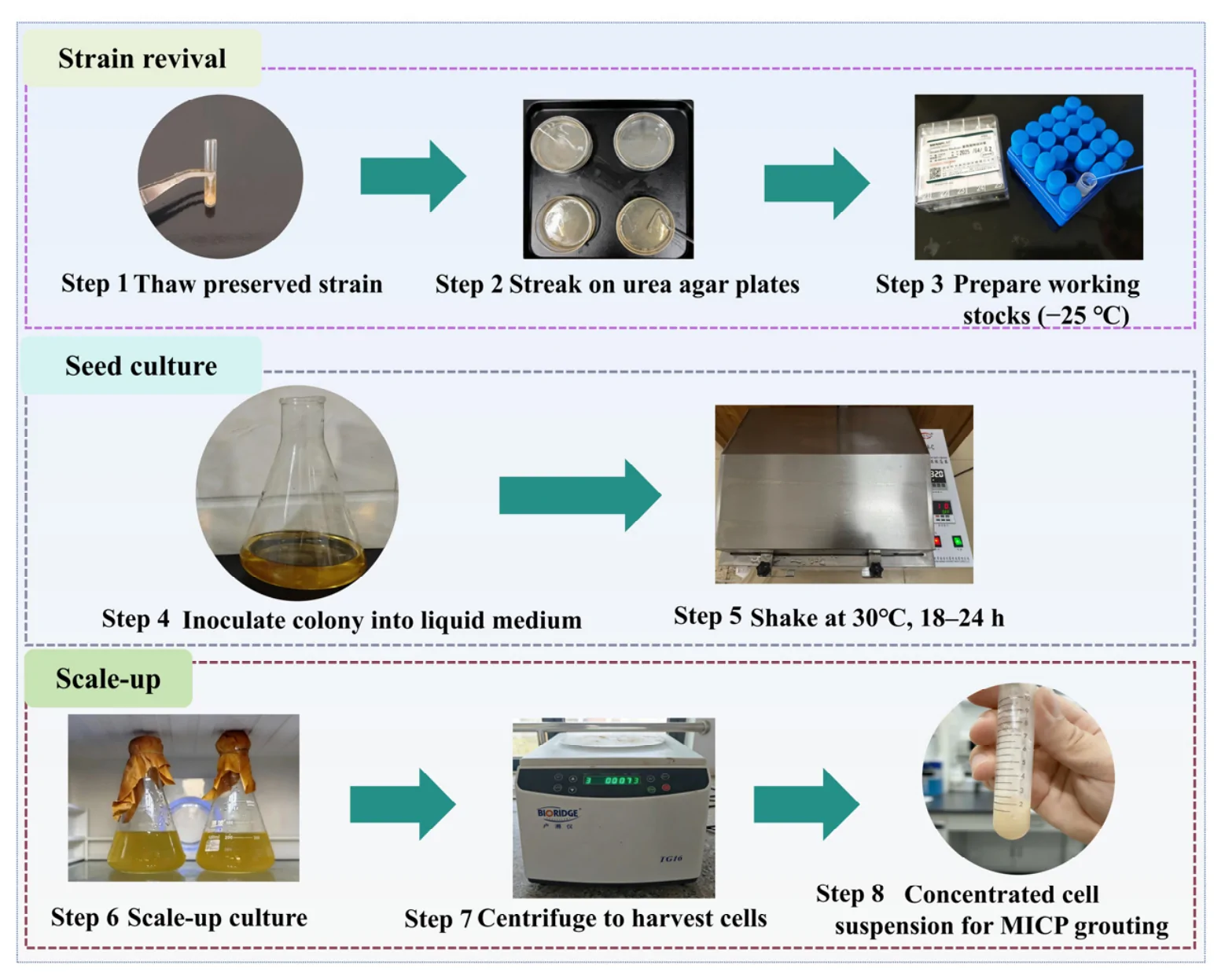
Flow chart of activation, preservation and scale-up cultivation of the urease-producing bacterium Sporosarcina pasteurii.

The procedures for bacterial cultivation, preparation of the working bacterial suspension, and scale-up cultivation are shown in Figure 5. During the activation stage, the lyophilized bacterial powder was reconstituted and spread onto a urea-containing solid medium, followed by aerobic incubation at 37.3 °C for 48 h to obtain typical single colonies. Subsequently, a single colony was picked to prepare cryovials, which were stored at −25 °C for later use [31]. During scale-up cultivation, approximately 20 ceramic beads from the cryovial were inoculated into 250 mL of sterilized liquid medium and cultured at 30 °C and 150 r/min for 24 h as the seed culture. The cells were then collected by centrifugation and incubated on a solid medium to form a dense bacterial lawn. After scraping, the bacterial lawn was inoculated into 1000 mL of sterilized liquid medium and further cultured under the same shaking conditions to obtain the bacterial suspension used for MICP grouting. During cultivation, OD600 was periodically measured to monitor bacterial growth status and the quality of the suspension.

To obtain a highly active bacterial suspension, bacterial growth and urease activity were monitored simultaneously. During 0–40 h of cultivation, samples were collected every 2 h to measure the optical density (OD600) and urease activity of the culture. The bacterial concentration was estimated by measuring OD600 at 600 nm using a UV–Vis spectrophotometer (X-7S, Shanghai Guangxi Instrument Co., Ltd., Shanghai, China), and the OD values were converted to the cell number concentration, Y (cells·mL^−1^), using Equation (1) [32]:

(1) *Y* = 8.59 × 10^7^ ×*Z*^1.3627^

where *Z* is the measured OD600 value.

Urease activity was determined using a conductivity method. Specifically, 6 mL of bacterial suspension was mixed with 54 mL of working urea solution and maintained at a constant temperature. The change in conductivity was recorded for 5 min, and the conductivity increase rate, K (μS·cm^−1^·min^−1^), was calculated. Urease activity was calculated according to the conversion proposed by Whiffin [33] (Equation (2)):

(2) *U* = 10 × *KA*

where *U* is the urea hydrolysis rate of the bacterial suspension, used to represent urease activity; *A* is an empirical conversion factor relating the change in conductivity to the amount of urea degraded, accounting for factors such as the reaction volume and temperature. The coefficient of 10 represents the dilution factor (60/6) and was used to convert the hydrolysis rate measured for 6 mL of bacterial suspension in a 60 mL reaction system into the urease activity of the original bacterial suspension.

Microbial activity within the tailings was further examined at the beginning of treatment, at the end of the 7 d cyclic grout-immersion treatment, and after the subsequent 7 d constant-temperature curing period. Urease activity in the tailings extract was determined using the conductivity method described above. Viable ureolytic bacterial abundance in the outer and inner regions of the specimens was determined using the plate-counting method and expressed as log_10_(CFU·g^−1^ dry tailings). Non-inoculated tailings maintained under the same conditions were also examined to evaluate the background contribution of indigenous microorganisms.

For plate counting, 5 g of fresh tailings was mixed with 20 mL of sterile 0.9% saline and serially diluted. An aliquot of 0.1 mL from the appropriate dilution was spread onto nutrient agar supplemented with 2% urea and incubated at 30 °C for 48 h. Three parallel samples were measured for each condition, and the results are expressed as the mean ± standard deviation.

**Figure 6:**
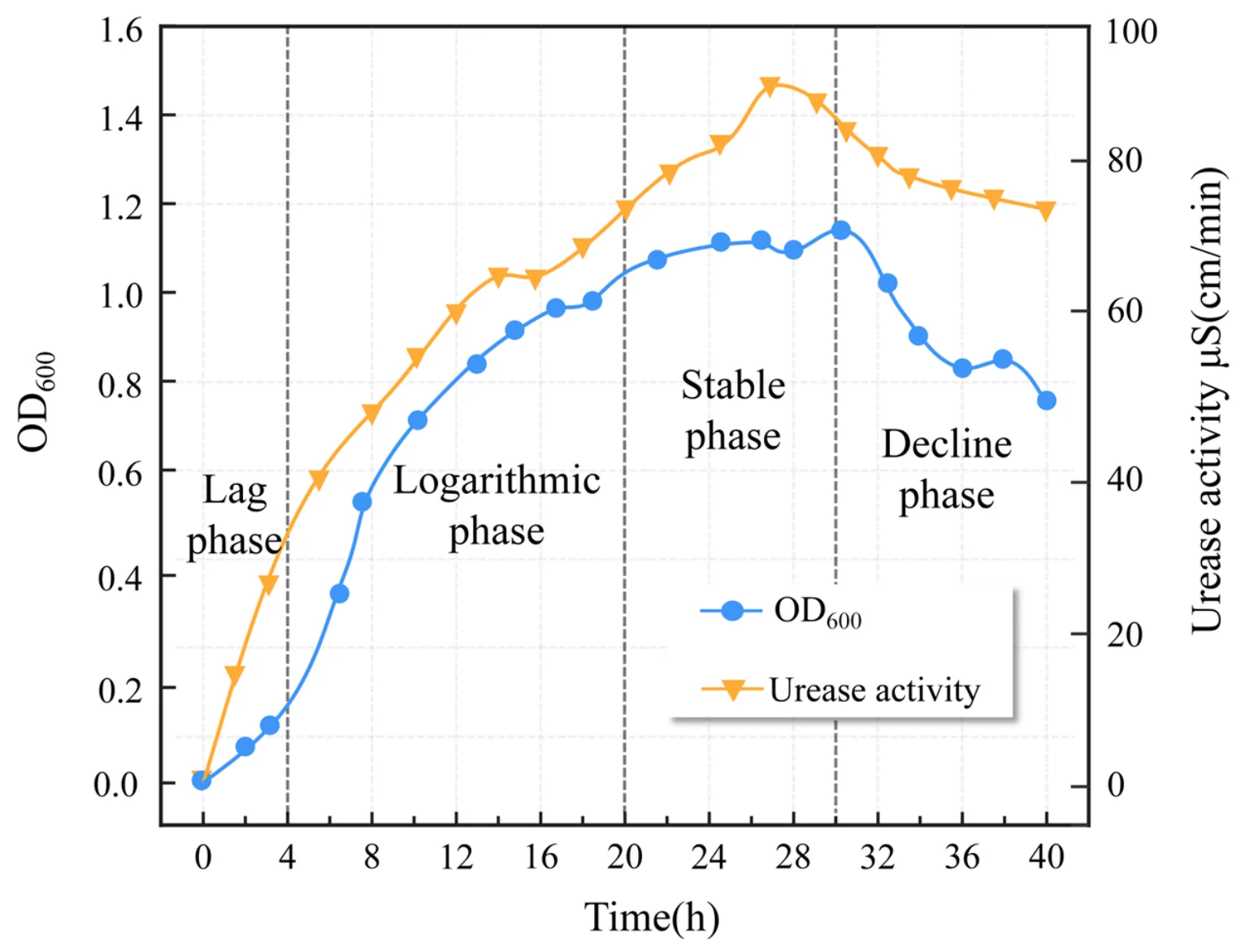
Growth and urease activity of Sporosarcina pasteurii during 40 h of cultivation. OD_600_ represents the optical density measured at 600 nm, and urease activity is expressed as the conductivity increase rate (μS·cm^−1^·min^−1^).

As shown in Figure 6, the cultivation process of Sporosarcina pasteurii comprised a lag phase at 0–4 h, an exponential phase at 4–20 h, a stationary phase at 20–30 h, and a subsequent decline phase at 30–40 h. The OD_600_ increased rapidly during the exponential phase and remained at approximately 1.09–1.14 during the stationary phase. Urease activity increased concurrently and reached a maximum of approximately 92 μS·cm^−1^·min^−1^ at approximately 26 h, after which it gradually decreased [34]. The culture collected at approximately 26 h was therefore used for subsequent MICP treatment because it combined a high cell density with maximum urease activity.

After the bacterial suspension was introduced into the tailings, urease activity gradually decreased but remained detectable throughout the treatment and curing periods. Approximately 45% of the initial activity remained at the end of the 7 d cyclic grout-immersion treatment, while approximately 19% remained after the subsequent 7 d curing period.

Viable ureolytic bacteria were detected in both the outer and inner regions of the specimens. The bacterial abundance decreased with time but remained measurable on Day 14. The abundance in the outer region was consistently higher than that in the inner region, with outer-to-inner ratios ranging from approximately 1.1 to 1.7. This moderate spatial gradient indicates preferential bacterial retention near the specimen surface, while also confirming that microbial activity was not restricted to a thin outer layer.

The non-inoculated tailings exhibited a relatively low background level of microbial abundance and urease activity. This comparison indicates that indigenous microorganisms may have made a limited contribution, whereas the introduced *S. pasteurii* was the main contributor to urea hydrolysis under the present treatment conditions. The detailed results are provided in Table 5.

#### 2.2.2. Specimen Preparation and Treatment Procedures

**Figure 7:**
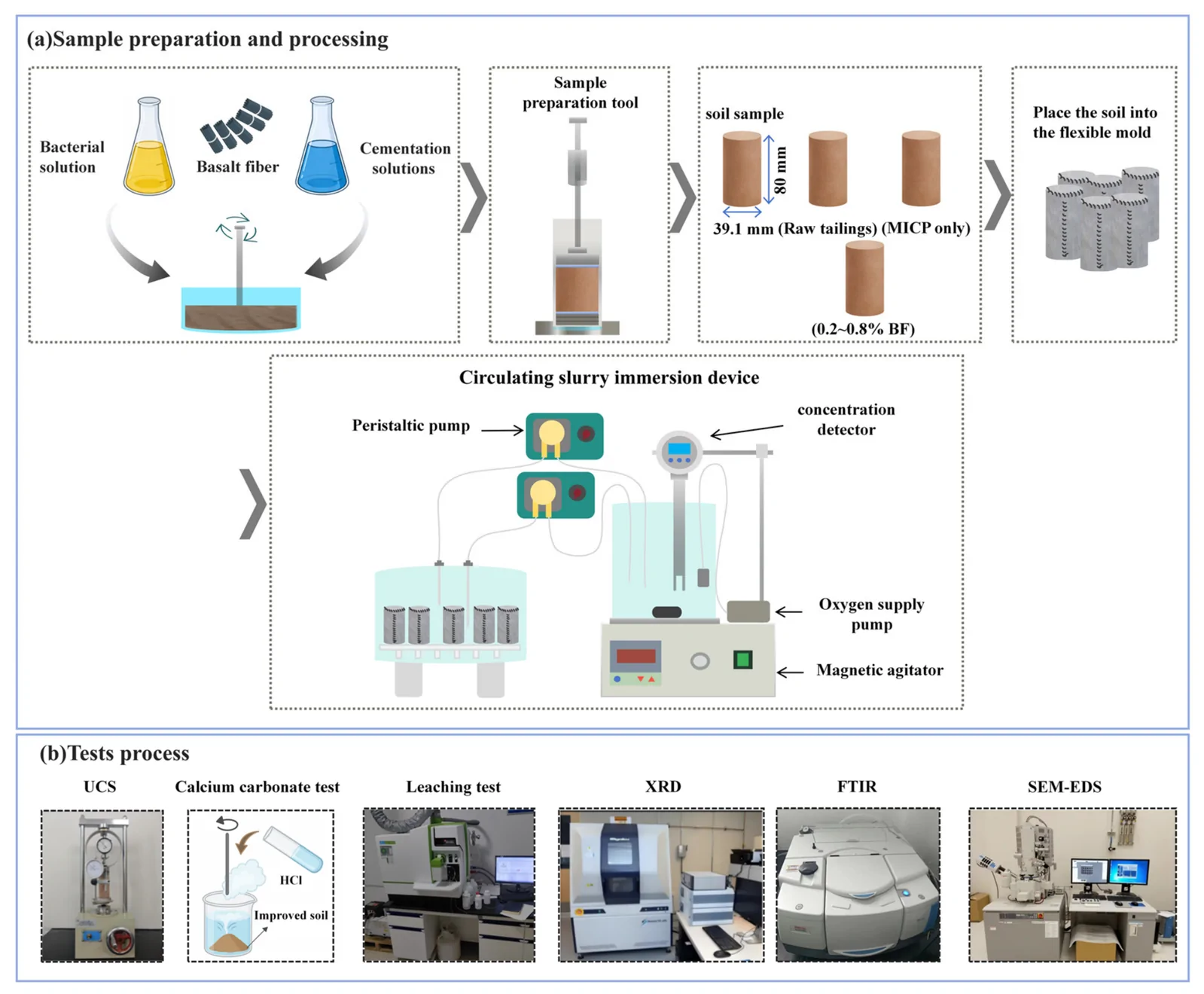
Preparation, treatment, and testing procedures for basalt-fiber-reinforced microbially induced carbonate precipitation (BF-MICP)-treated tailings specimens: (**a**) specimen preparation and cyclic grout-immersion treatment; (**b**) unconfined compressive strength (UCS), calcium carbonate content, leaching, X-ray diffraction (XRD), Fourier-transform infrared spectroscopy (FTIR), and scanning electron microscopy with energy-dispersive spectroscopy (SEM–EDS) tests.

The previously prepared Sporosarcina pasteurii suspension was used to conduct MICP treatment on basalt-fiber-reinforced ion-type rare earth tailings soil. The specimen preparation procedure is shown in Figure 7. First, the air-dried and sieved tailings soil was dry-mixed with basalt fiber at predetermined fiber dosage (0, 0.2%, 0.3%, 0.4%, 0.6%, and 0.8%) to ensure adequate dispersion of fibers within the soil. The upper dosage limit of 0.8% was determined based on preliminary tests, which showed that further increasing the basalt fiber content led to more obvious fiber agglomeration, poorer mixture workability and specimen-forming quality, and no further improvement in reinforcement effectiveness. Therefore, 0.8% was selected as the upper bound to cover the transition from effective fiber addition to the onset of adverse overdosage effects. Dry mixing was used to improve fiber dispersion and reduce the likelihood of large-scale bundling. However, owing to the subsequent layered compaction, a certain preference for sub-horizontal fiber orientation may have developed. Subsequently, the bacterial suspension prepared at the prescribed ratio was added, and the mixture was mechanically stirred until a homogeneous slurry was obtained. Thus, the bacterial suspension was initially distributed throughout the tailings before compaction rather than being introduced only from the outer surface of the specimen.

The specimens included an untreated tailings group (raw tailings), an MICP-only group (0% fiber), and BF-MICP groups with different fiber dosages. The grouping and coding of specimens are presented in Table 6. At a representative basalt fiber content of 0.4%, two control groups were included to distinguish the contribution of active bacterial treatment from non-biological effects. In the 0.4 BF-SM group, the active bacterial suspension was replaced with an equal volume of sterile culture medium. In the 0.4 BF-KB group, a heat-inactivated bacterial suspension prepared at the same initial OD_600_ was used. All other specimen preparation, cementation-solution circulation, and curing conditions were identical to those of the 0.4 BF-MICP group. The same codes were used throughout the subsequent figures and tables. Except for the untreated, 0.8 BF-only, 0.4 BF-SM, and 0.4 BF-KB groups, all MICP-treated dosage groups followed the same treatment procedure, with basalt fiber dosage as the only variable. The mixture was compacted into cylindrical molds in layers to prepare specimens with dimensions of 39.1 mm in diameter and 80 mm in height.

After molding, each specimen was transferred into a flexible cylindrical geotextile mold to reduce particle loss during treatment. The flexible mold had dimensions of 39.4 mm in diameter and 83 mm in height. Before constant-temperature curing, a cyclic grout-immersion method was applied [35]. During this process, the cementation solution was circulated through the reactor using a peristaltic pump at a rate of 1 mL/min, and one complete grout circulation was achieved in approximately 3 d. The cyclic grout-immersion treatment was continuously maintained for 7 d, after which the specimens were removed from the flexible molds and cured under constant-temperature conditions for another 7 d before subsequent tests were conducted. Three parallel specimens were prepared for each test condition.

#### 2.2.3. Unconfined Compressive Strength and Toxicity Characteristic Leaching Tests

A YYW-2 strain-controlled unconfined compression apparatus was used to evaluate the mechanical enhancement of tailings soil treated by BF-MICP, with untreated soil and the MICP-only group serving as controls. Cylindrical specimens (39.1 mm in diameter and 80.0 mm in height) were tested after 7 d of curing under constant-temperature conditions following cyclic grout immersion. Loading was applied at a rate of 1.0 mm/min, and each test was completed within 8–20 min. Peak stress, stress–strain curves, and typical failure modes were recorded. For each condition, three parallel specimens were prepared for UCS testing.

The toxicity characteristic leaching procedure (TCLP) followed HJ/T 300-2007 [36] (Solid waste—Leaching toxicity leaching method—Acetic acid buffer solution method) and US EPA SW-846 Method 1311 (TCLP). Specimens at the same curing age (7 d) were collected, oven-dried, crushed, and sieved, and then extracted using an acetic acid buffer solution. The supernatant was separated to determine the leached concentrations of Pb and Zn, and the leached concentrations together with their relative reductions were used to characterize the stabilization performance. The speciation of Pb and Zn was determined using a modified BCR sequential extraction procedure, in which the metals were fractionated into acid-extractable, reducible, oxidizable, and residual fractions. Three parallel specimens were prepared for the untreated group and each treated group.

#### 2.2.4. Determination of CaCO_3_ Production

To quantitatively determine the CaCO_3_ content produced by MICP, an acid-leaching mass-loss method was used to measure the carbonate production rate [37]. The treated tailings specimens were gently ground and sieved. Representative subsamples were oven-dried at 105 °C to constant mass, and the mass before acid leaching was recorded as *m*_1_. The subsamples were then repeatedly soaked in 1 mol/L HCl with gentle shaking until CO_2_ bubbles were no longer observed on the specimen surface. After leaching, the subsamples were thoroughly rinsed with deionized water to remove residual acid and soluble salts, and then oven-dried again at 105 °C to constant mass. The mass after acid leaching was recorded as *m*_2_. The mass difference between the two constant-mass measurements (*m*_1_–*m*_2_) corresponds to the carbonate mass removed by acid dissolution and was taken as the CaCO_3_ content produced by MICP. The carbonate production rate, η, was expressed as a mass fraction and calculated using Equation (3). For each condition, three parallel specimens were tested.

(3) $$\eta =\frac{\left(m_{1}-m_{2}\right)}{m_{1}}\times 100\%$$

#### 2.2.5. Microstructural and Mineralogical Characterization

To characterize the microstructural evolution of the tailings framework and the stabilization of Pb^2+^ and Zn^2+^ under BF-MICP treatment, micro-analyses were performed using SEM-EDS, XRD, and FTIR. Representative specimens were selected for SEM observation of pore structure, CaCO_3_ precipitation morphology, and fiber–particle interfacial features. Elemental distributions of Ca, Pb, and Zn in interfacial regions were obtained by EDS. XRD (Cu-Kα radiation, 2θ = 10–70°) was used to identify CaCO_3_ and major silicate mineral phases and to compare changes before and after treatment. FTIR (KBr pellets, 4000–400 cm^−1^) was employed to analyze characteristic absorption bands of carbonate functional groups and the Si-O framework, providing molecular-level evidence for the mechanistic discussion [38,39].

#### 2.2.6. Statistical Analysis

Quantitative results are presented as the mean ± standard deviation (SD) of three parallel specimens. Statistical analysis was performed using Origin 2021 (OriginLab Corporation, Northampton, MA, USA). Differences among the treatment groups were evaluated by one-way analysis of variance (ANOVA), followed by Tukey’s multiple-comparison test. Differences were considered statistically significant at *p* < 0.05.

## 3. Results and Discussion

### 3.1. Strength and Energy Characteristics

#### 3.1.1. Unconfined Compressive Strength

**Figure 8:**
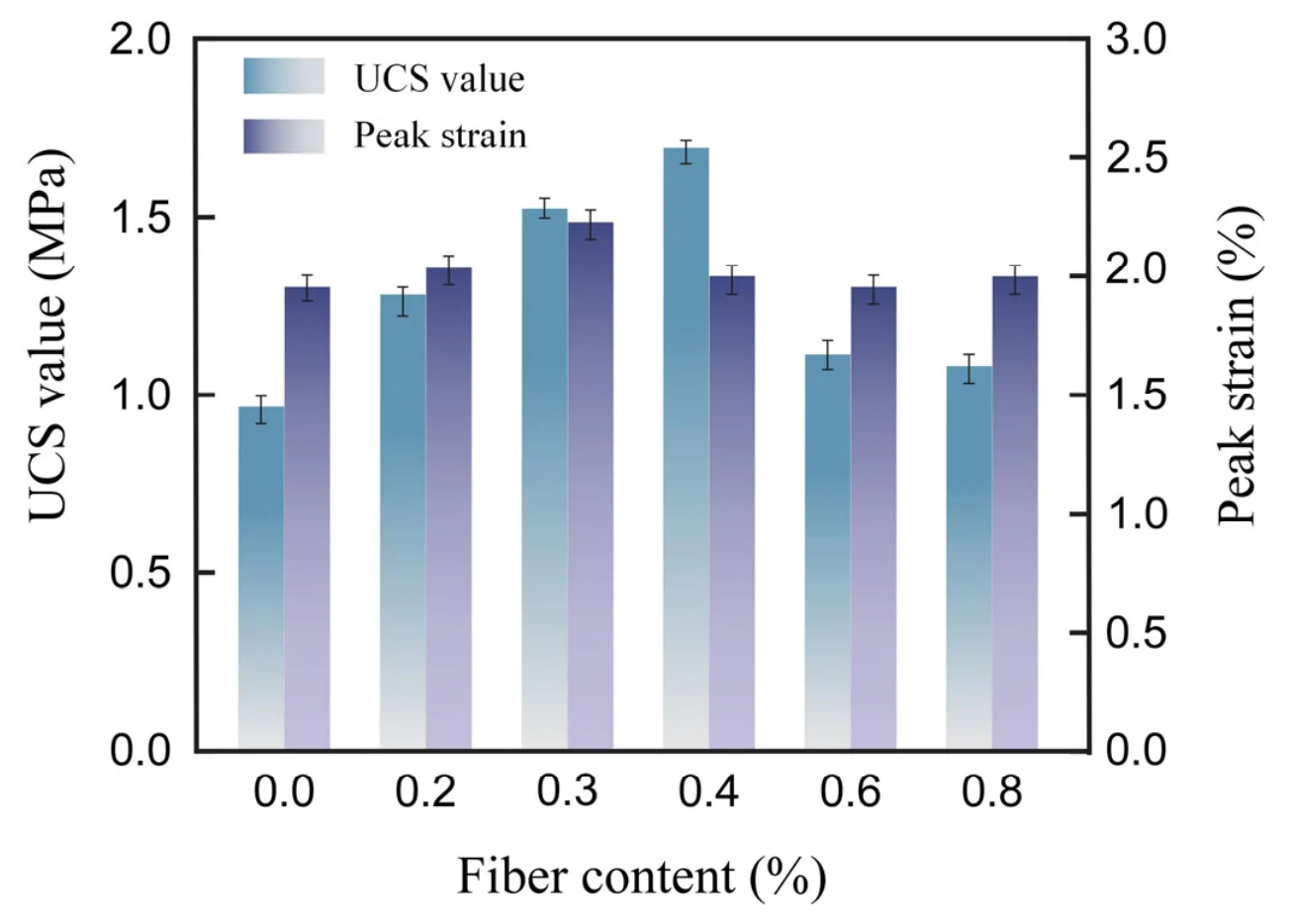
Unconfined compressive strength and corresponding peak strain of BF-MICP-treated tailings at different basalt fiber contents. Error bars represent the standard deviation of three parallel specimens.

The solidification/stabilization performance of BF-MICP for ion-type rare earth tailings was evaluated using unconfined compressive strength (UCS). According to relevant requirements of the U.S. Environmental Protection Agency (US EPA), the UCS of solidified soils should generally be no lower than 350 kPa [40]. Figure 8 presents the UCS and peak strain of BF-MICP-solidified tailings at different basalt fiber contents. For the fiber-free group (0.0 BF-MICP), the UCS was 0.91 MPa, the peak strain was approximately 1.8%, and the specimens exhibited predominantly brittle crushing failure. Low fiber dosages (0.2–0.3%) markedly improved the mechanical performance: the UCS increased to 1.33 and 1.51 MPa, representing increases of 46% and 66% relative to the 0.0 BF + MICP group, while the peak strain simultaneously rose to 2.1% and 2.4%, respectively. This indicates that, at this stage, the fibers provided effective bridging within the CaCO_3_-cemented framework by transferring tensile forces and restraining crack propagation. Consequently, the ultimate load-bearing capacity increased, and the specimens developed a certain degree of plastic deformability.

When the fiber content was further increased to 0.4%, the UCS reached a maximum of 1.72 MPa, which was 90% higher than that of the fiber-free group. The peak strain was approximately 2.1%, indicating the optimal strength performance at this dosage without an obvious reduction in pre-peak deformability. When the fiber content increased to 0.6% and 0.8%, the UCS decreased to 1.21 and 1.17 MPa, respectively, and the peak strain dropped to 1.7–2.0%. These results suggest that excessive fiber reduces the reinforcement efficiency; at high dosages, fibers are more prone to agglomeration, thereby increasing interfacial defects. Locally, elevated porosity or insufficient CaCO_3_ deposition may occur, forming weakened zones that promote stress concentration and lead to premature failure [41]. One-way ANOVA showed that fiber dosage had a significant effect on UCS (*p* < 0.001). Tukey’s test indicated that the UCS of the 0.4 BF-MICP group was significantly higher than that of the 0.0 BF-MICP group (*p* < 0.001). Overall, the UCS first increased and then decreased with increasing fiber content, and fiber contents of 0.3–0.4% provided a more favorable overall mechanical response. It should be noted that layered compaction may have produced a certain preference for sub-horizontal fiber orientation, approximately parallel to the compaction planes. Under axial compression, fibers with such an orientation may restrict lateral deformation and provide bridging and pull-out resistance across axial-splitting and inclined shear cracks, thereby contributing to the observed strength and post-peak performance. Because all specimens were prepared using the same mixing and compaction procedures and tested under the same axial loading condition, the comparative trends among different fiber contents remain meaningful under the present test conditions. The exact fiber-orientation distribution was not quantitatively characterized, and its influence under different loading directions and triaxial stress states requires further investigation.

#### 3.1.2. Stress–Strain Response and Failure Characteristics

**Figure 9:**
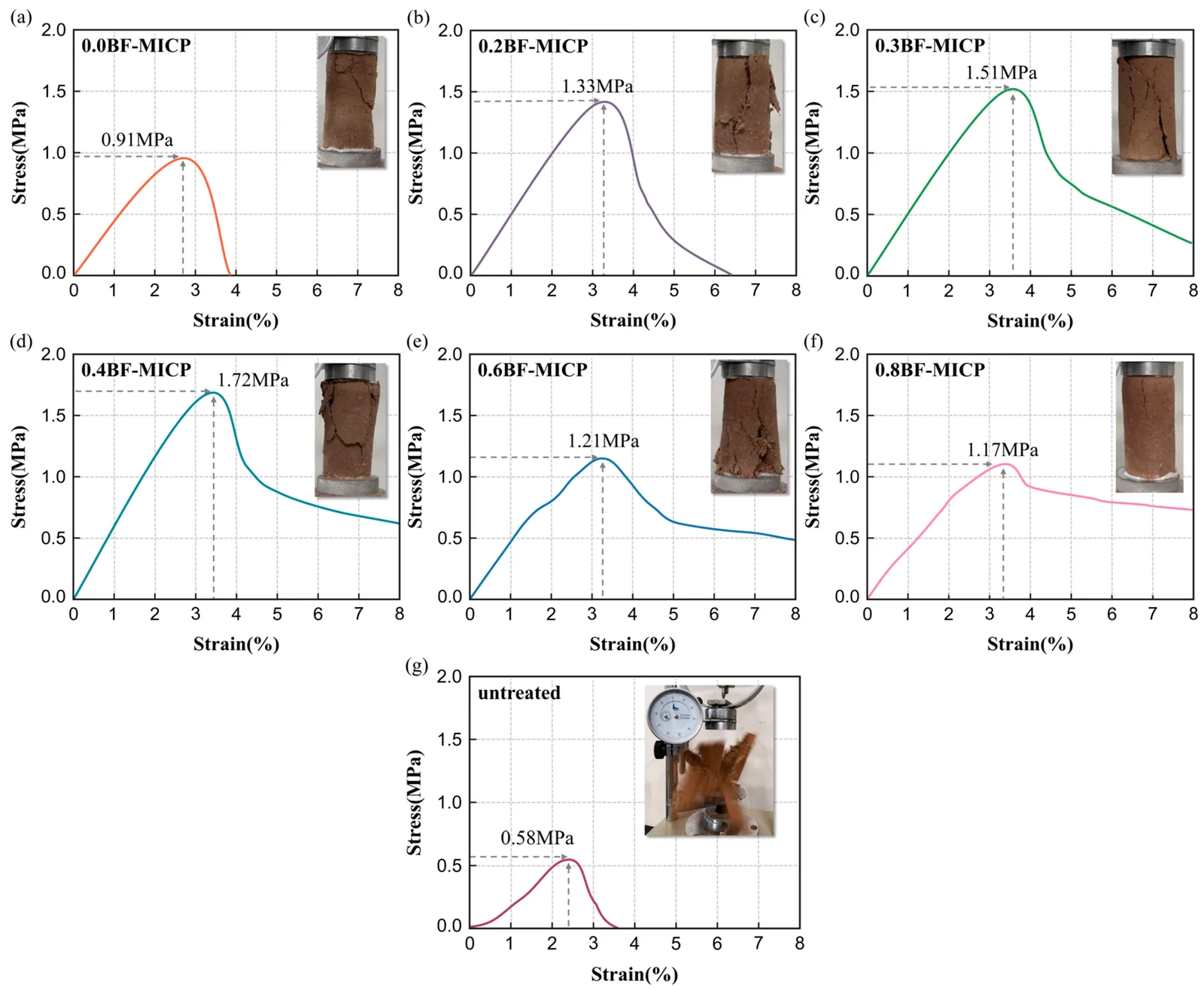
Representative axial stress–strain curves and typical failure modes of tailings specimens under different treatment conditions: (**a**) 0.0 BF-MICP; (**b**) 0.2 BF-MICP; (**c**) 0.3 BF-MICP; (**d**) 0.4 BF-MICP; (**e**) 0.6 BF-MICP; (**f**) 0.8 BF-MICP; and (**g**) untreated tailings. The numerical value preceding “BF-MICP” denotes the basalt fiber content by dry mass of tailings.

Figure 9 shows the stress–strain curves and typical failure patterns of rare earth tailings treated by MICP under different basalt fiber dosages. The untreated tailings exhibited a peak strength of only 0.58 MPa with a small peak strain. After reaching the peak, the stress dropped rapidly, and the specimen disintegrated instantaneously into multiple fragments under impact-shear actions, indicating a typical strongly brittle failure behavior [42]. For the 0.0 BF + MICP group (MICP only), the peak strength increased to approximately 0.91 MPa and the peak strain increased slightly; however, the post-peak stress decayed rapidly, showing pronounced brittle softening. A major crack developed along the lateral surface, with branching cracks near the upper part. Minor spalling and particle shedding were observed locally, and the overall failure was dominated by splitting. These observations indicate that CaCO_3_ cementation can substantially enhance load-bearing capacity, but its contribution to improving overall ductility is limited.

As the basalt fiber dosage increased from 0.2% to 0.4%, the synergistic reinforcement effect of BF-MICP became evident: the peak strength of the 0.2 BF-MICP and 0.3 BF-MICP groups increased to 1.33 and 1.51 MPa, respectively. Both the peak strain and ultimate strain increased markedly, and the post-peak stress decay was significantly slowed. Correspondingly, surface cracking evolved from a single vertical crack to a combined pattern governed by inclined shear cracks and vertical cracks. In addition, crack paths became rough and tortuous rather than straight, and a “crack band” formed locally due to the interweaving of multiple fine cracks. At a fiber dosage of 0.4%, the peak strength reached 1.72 MPa, the highest among all groups. The stress–strain curve exhibited a relatively smooth peak, and the post-peak strength decreased more gradually. Although extensive surface spalling occurred and numerous cracks developed, the specimen remained overall intact. These results suggest that an appropriate amount of basalt fiber, together with CaCO_3_ bridging, formed a continuous and stable three-dimensional load-bearing framework.

When the fiber dosage increased to 0.6%, the peak strength decreased from 1.72 MPa (0.4% group) to 1.21 MPa. Post-peak softening was partially alleviated, and multiple fine inclined cracks were observed on the lateral surface, accompanied by slight bulging in local areas. With a further increase to 0.8%, the peak strength dropped to 1.17 MPa. The post-peak segment became smoother, but the overall post-peak load-bearing level remained low. The overall outline of the specimen remained largely intact, with only one or two narrow through-cracks, and the failure process was relatively more gradual. This indicates that basalt fiber at dosages of 0.3–0.4% can simultaneously improve peak strength and ductility. When the dosage was further increased, fibers became more prone to agglomeration and may form localized weak zones, leading to a slight reduction in peak strength.

#### 3.1.3. Residual Stress and Energy Dissipation

In this study, the stress corresponding to an additional 3% axial strain beyond the peak strain was adopted as the residual stress. [43]. A higher residual stress indicates that a certain load-bearing capacity can still be maintained after the peak, implying a lower tendency toward brittle instability and better post-peak ductility (toughness). Residual stress is commonly used to characterize the load-bearing level that a specimen can sustain after failure. The energy absorbed during uniaxial compression was taken as the area under the stress–strain curve from the loading origin to the residual-stress point, a definition that has been widely adopted in related studies such as fiber-reinforced concrete [44]. The CTE values were calculated from the original stress–strain data by numerical integration and were used to quantify the energy dissipation capacity of BF-MICP specimens during failure. This area was defined as the compressive total energy absorption (CTE) and was used to quantify the energy dissipation capacity of BF-MICP specimens during failure. A higher CTE indicates that more externally input energy can be absorbed and dissipated during the failure process, thereby reflecting better toughness and post-peak deformation capacity.

**Figure 10:**
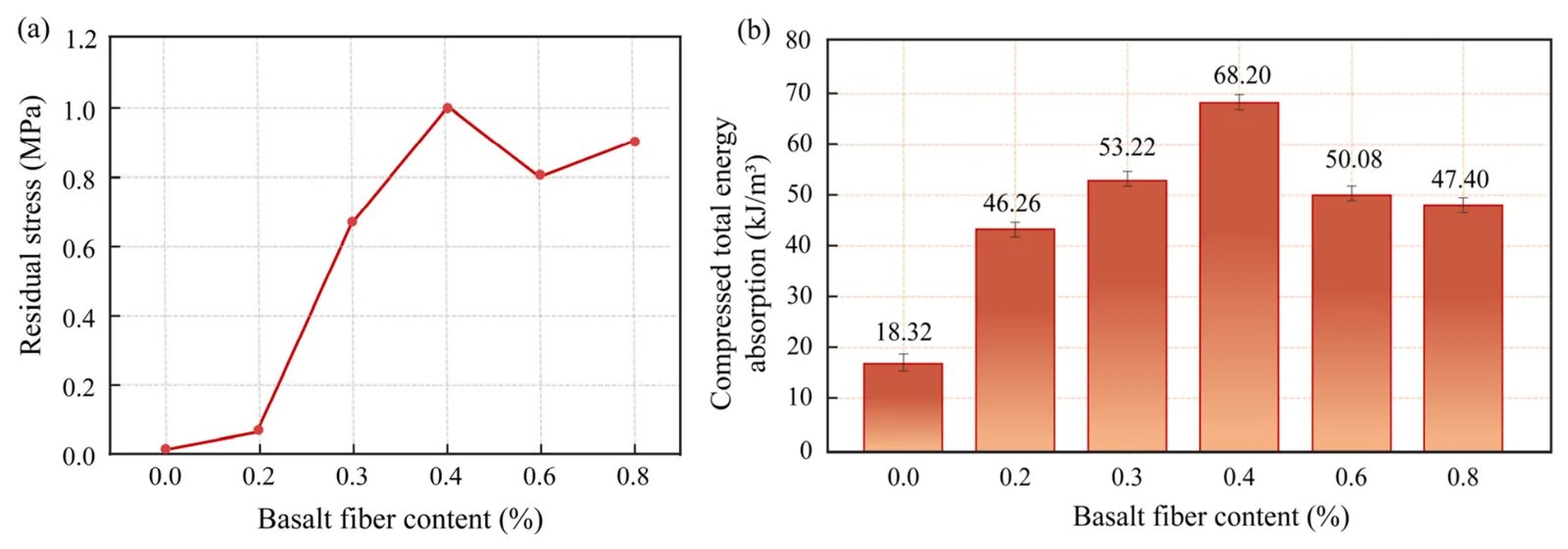
Post-peak mechanical characteristics of BF-MICP-treated tailings at different basalt fiber contents: (**a**) residual stress determined at an additional axial strain of 3% beyond the peak strain; and (**b**) compressive total energy absorption (CTE). Error bars in panel (**b**) represent the standard deviation of three parallel specimens.

Figure 10 presents the residual stress and compressive total energy absorption (CTE) of BF-MICP-treated tailings with different basalt fiber dosages. As shown in Figure 10a, residual stress was more sensitive to fiber dosage. Without fibers, the residual stress was nearly zero, and the post-peak load-bearing capacity was very limited, indicating that CaCO_3_ cementation alone still exhibited predominantly brittle instability. When the fiber dosage increased from 0.2% to 0.3% and 0.4%, the residual stress rose rapidly and reached its maximum at 0.4%, showing an order-of-magnitude increase relative to the fiber-free group; accordingly, the post-peak bearing capacity and ductility were improved most evidently. With further increases to 0.6% and 0.8%, the residual stress decreased slightly but remained at a relatively high level, suggesting that peak strength may be constrained at high dosages, whereas the structure did not rapidly lose its load-bearing capacity after failure. Considering the curve shapes and observed failure features, it is inferred that, in the post-peak stage, fibers undergo stretching, pullout, and interfacial friction near the failure plane, suppressing crack opening and sliding and thereby delaying disintegration while retaining residual capacity.

As shown in Figure 10b, CTE increased continuously as the fiber dosage rose from 0 to 0.4%, from approximately 18 kJ/m^3^ in the fiber-free group to about 68 kJ/m^3^ at 0.4% BF. Fiber dosage had a significant effect on CTE (*p* < 0.001), and the CTE of the 0.4 BF-MICP group was significantly higher than that of the 0.0 BF-MICP group according to Tukey’s test (*p* < 0.001). This indicates that the incorporation of basalt fiber markedly enhanced the energy dissipation capacity of the BF-MICP-treated tailings within the low-to-moderate dosage range. With further increases to 0.6% and 0.8%, CTE decreased slightly but remained substantially higher than that of the fiber-free group, suggesting that although excessive fiber addition weakened the reinforcement effect at the peak stage, it still contributed to post-peak energy dissipation and residual load-bearing capacity. Considering both residual stress and CTE, a fiber dosage of 0.3–0.4% is more favorable for improving post-peak bearing capacity and toughness of BF-MICP-solidified tailings, consistent with the optimal fiber range identified from the UCS results.

### 3.2. TCLP Leaching Behavior

**Figure 11:**
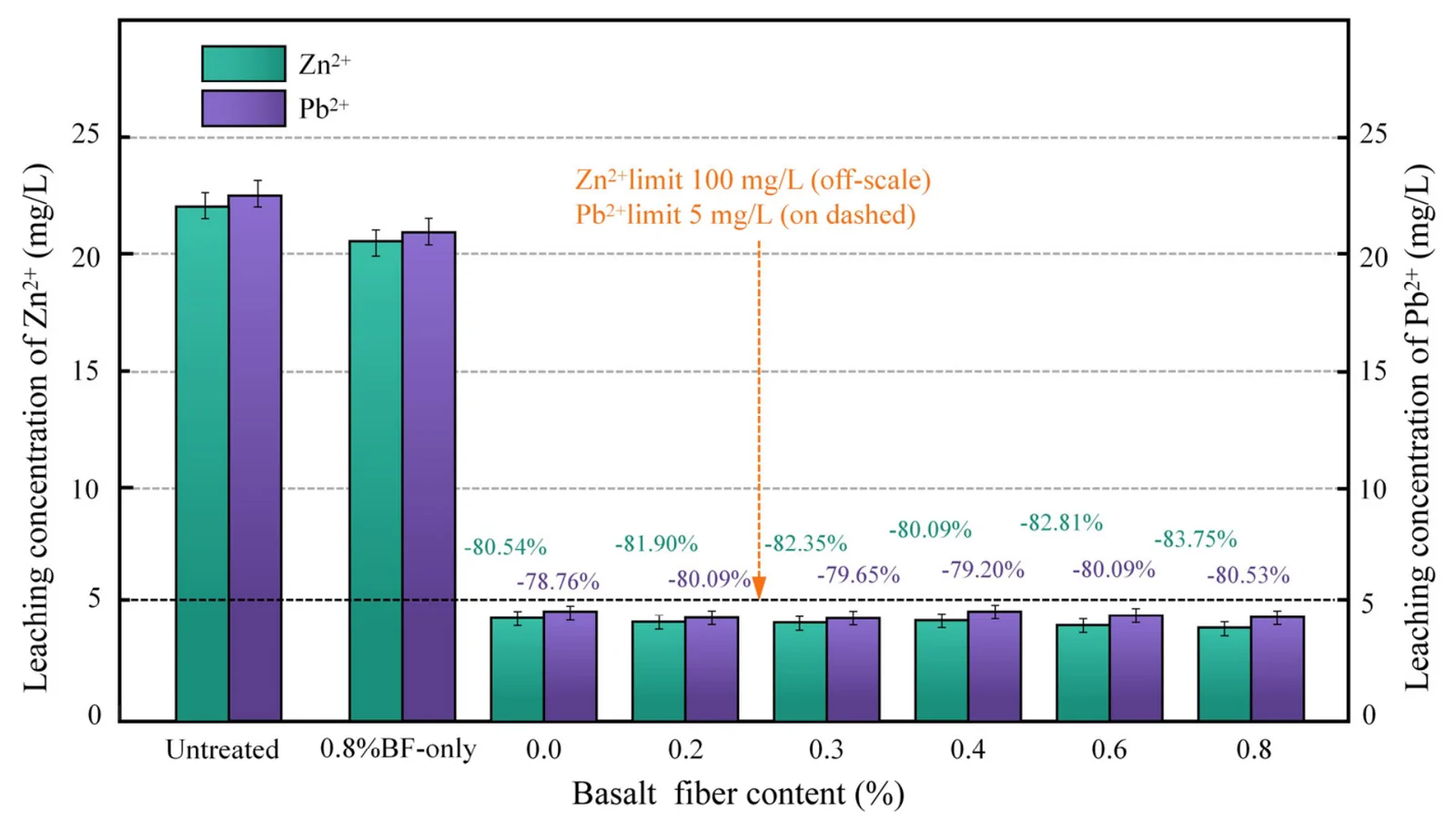
Toxicity characteristic leaching procedure (TCLP) concentrations of Pb^2+^ and Zn^2+^ in tailings under different treatment conditions. Percentages above the bars indicate the reductions in leached concentrations relative to the untreated group. Error bars represent the standard deviation of three parallel specimens. The horizontal dashed line indicates the regulatory limit for Pb^2+^ of 5 mg·L^−1^; the Zn^2+^ limit of 100 mg·L^−1^ is outside the plotted range.

Residual Pb and Zn in ion-type rare earth tailings can be readily leached and transported to surrounding soils and water bodies under rainfall and long-term infiltration of surface water and groundwater [45]. For BF-MICP-treated tailings, the concentrations of Pb^2+^ and Zn^2+^ in the TCLP leachate are key indicators for assessing solidification/stabilization performance and environmental safety. The leaching toxicity under different treatment conditions was evaluated with reference to GB 5085.3—2007 [46] (Pb^2+^ ≤ 5 mg/L; Zn^2+^ ≤ 100 mg/L). To distinguish the effects of fiber addition alone from those of biomineralization-induced cementation, an additional 0.8 BF-only group (0.8% basalt fiber without MICP treatment) was included as a TCLP control. This group was used only for the leaching tests. The variations in Pb and Zn concentrations among different groups are shown in Figure 11.

In this study, the initial mass fractions of Pb and Zn in the soil samples were both controlled at 500 mg/kg. For the untreated tailings, the leached concentrations of Pb and Zn were 22.6 mg/L and 22.1 mg/L, respectively; Pb exceeded the standard limit by approximately 4.5 times. This indicates a pronounced risk of releasing leachable heavy metals. For the 0.8%BF-only group, the leached concentrations of Pb and Zn were 20.9 mg/L and 20.5 mg/L, respectively, which were comparable to those of the untreated group and still far above the Pb limit. This suggests that fiber addition alone has little effect on reducing heavy-metal leaching. The variability among parallel samples was small across all groups, indicating good reproducibility of the leaching results.

In contrast, after BF-MICP solidification, the leached concentrations of Pb and Zn in all treated groups decreased markedly and were generally maintained at approximately 3.5–4.5 mg/L, all below the safety thresholds specified in GB 5085.3—2007. Relative to the untreated tailings, the Pb and Zn leached concentrations in the BF-MICP groups decreased by approximately 78–84% (percentages above the bars), indicating that microbially induced carbonate precipitation achieved effective immobilization of heavy metals in the tailings. One-way ANOVA showed that the treatment condition had a significant effect on the leached concentrations of both Pb and Zn (*p* < 0.001). Tukey’s test showed that the MICP-treated groups had significantly lower Pb and Zn concentrations than the untreated group (*p* < 0.001).

It is inferred that, during CaCO_3_ precipitation, Pb and Zn can co-precipitate with carbonates or be encapsulated by CaCO_3_, and may also be incorporated into the carbonate crystal lattice, thereby reducing their solubility and mobility [47]. CaCO_3_ deposited within pores and filled pore channels, increasing specimen densification and hindering leachate ingress and outward migration of heavy metals.

Among the MICP-treated groups, no significant differences in Pb or Zn leaching concentrations were observed with increasing fiber dosage (*p* > 0.05). This indicates that the dominant factors governing heavy-metal stabilization are still the amount of CaCO_3_ produced and its deposition mode, whereas the direct chemical effect of basalt fiber is limited. Basalt fiber primarily enhances the mechanical performance of the solidified matrix by constructing a load-bearing framework and altering crack propagation and failure modes. Under the short-duration acidic extraction conditions of TCLP, such structural improvements are difficult to translate into further decreases in leached concentrations. Therefore, the contribution of fibers to stabilization is generally indirect and limited.

### 3.3. Heavy Metal Speciation Characteristics

**Figure 12:**
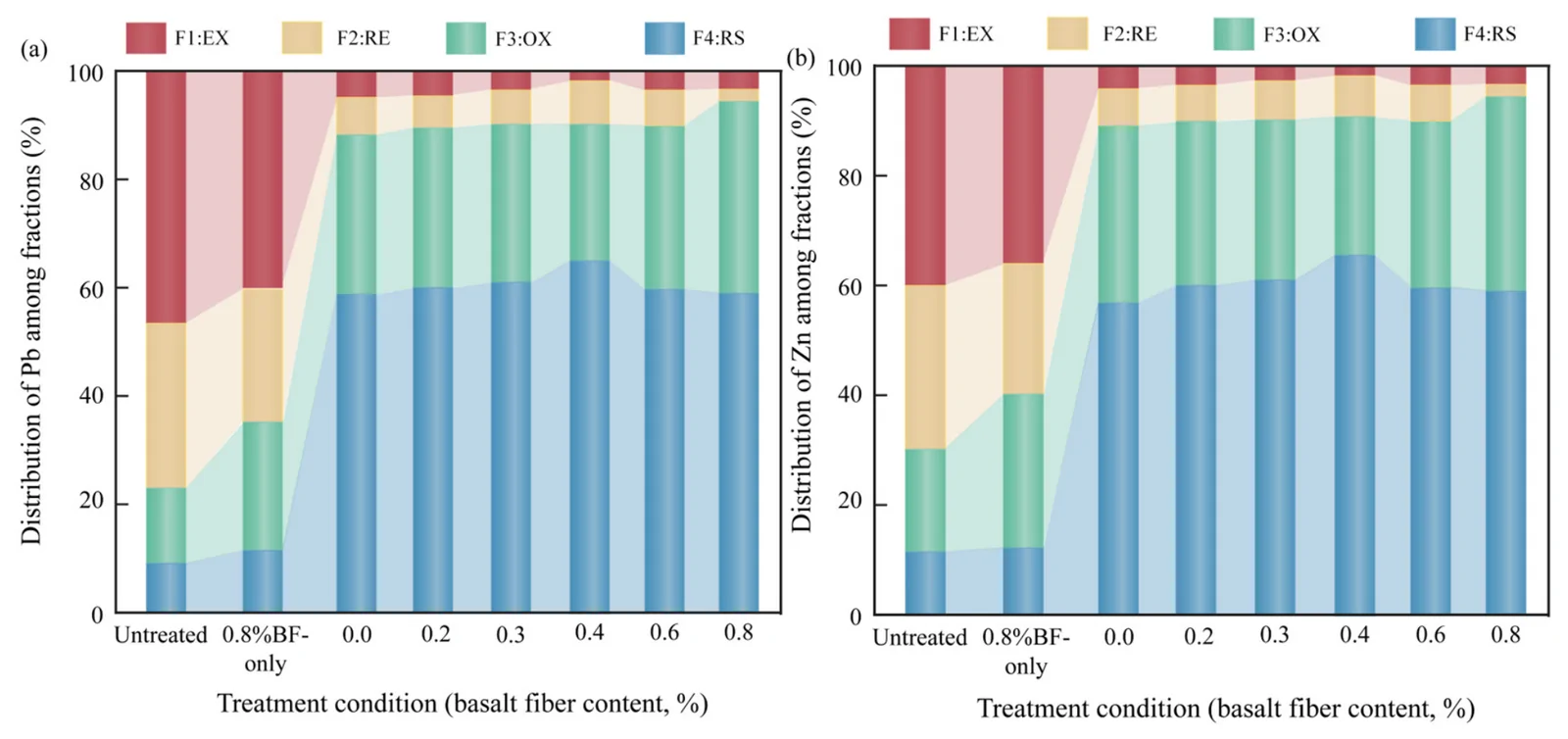
Distribution of Pb and Zn fractions determined using the modified Community Bureau of Reference (BCR) sequential extraction procedure: (**a**) Pb; (**b**) Zn. F1, acid-extractable fraction; F2, reducible fraction; F3, oxidizable fraction; F4, residual fraction.

To further elucidate the mechanisms by which BF-MICP stabilizes Pb and Zn in tailings, heavy-metal speciation was analyzed using a modified BCR sequential extraction procedure. According to the extraction sequence, Pb and Zn were fractionated into the acid-extractable fraction (F1), reducible fraction (F2), oxidizable fraction (F3), and residual fraction (F4) [48], among which F1 and F2 exhibit higher mobility and pose greater environmental risk, whereas F3 and F4 are relatively stable. The speciation distributions of Pb and Zn under different treatment conditions are shown in Figure 12.

In the untreated tailings, Pb was mainly present in F1 and F2, accounting for 45.83% and 30.27%, respectively, whereas the residual fraction (F4) accounted for only 7.94%. Zn showed a similar pattern, with F1 and F2 accounting for 40.12% and 29.88%, respectively, and F4 accounting for only 12.47%. These results indicate that Pb and Zn in the untreated tailings were dominated by highly mobile fractions, which is consistent with the relatively high leaching concentrations observed in the TCLP results. In the 0.8%BF-only group, in which basalt fiber was added without MICP treatment, F1 + F2 remained 64.29% for Pb and 59.14% for Zn, indicating that the high-risk fractions still predominated. This suggests that fiber addition alone exerts only a limited direct effect on regulating heavy-metal speciation.

After MICP treatment, the speciation of Pb and Zn changed markedly. Taking the 0.0 BF + MICP group as an example, Pb F1 decreased from 45.83% to 3.72% and F2 from 30.27% to 8.14%, while F3 and F4 increased to 30.18% and 57.96%, respectively, with a combined F3 + F4 of 88.14%. For Zn, F1 and F2 decreased to 3.21% and 8.07%, respectively, whereas F3 and F4 increased to 31.29% and 57.43%, respectively, giving a combined F3 + F4 of 88.72%. These results indicate that the CaCO_3_ produced by MICP can markedly reduce the labile fractions of Pb and Zn and promote their transformation into stable fractions, and is therefore the dominant factor governing heavy-metal stabilization.

Across the BF-MICP groups, the speciation transformation of Pb and Zn generally followed the same dominant pathway described above, namely from F1/F2 to F3/F4. Although the differences among fiber dosages were relatively limited, a discernible optimal dosage range was still observed. For Pb, F1 + F2 remained at a low level of 7.40–11.10% across the BF-MICP groups, corresponding to an F4 proportion of 57.93–65.02%. Among them, the 0.4 BF + MICP group showed the most favorable performance, with F1 = 1.83%, F1 + F2 = 7.40%, and F4 = 65.02%. When the fiber dosage increased further to 0.6–0.8%, F1 + F2 increased slightly (10.31–10.67%) and F4 decreased slightly (59.84–57.93%), suggesting that the marginal benefit of speciation optimization weakened at high fiber dosages. For Zn, F1 + F2 ranged from 7.07% to 10.63% and F4 from 58.35% to 65.57% across the BF-MICP groups, with the optimum again observed at 0.4 BF + MICP (F1 + F2 = 7.07%, F4 = 65.57%).

Taken together, the results for Pb and Zn show a highly consistent evolution pattern after BF-MICP treatment: the highly mobile fractions decreased markedly, whereas the stable fractions increased substantially. Combined with the TCLP results, the pronounced reductions in Pb and Zn leaching concentrations can be attributed mainly to the combined effects of CaCO_3_ encapsulation, pore-structure densification, and fraction transformation, whereas the contribution of basalt fiber to speciation optimization was more likely reflected in improving the spatial distribution of biomineralization products and the interfacial effects within a moderate dosage range. When the fiber dosage was further increased, this effect was no longer evident. This trend is consistent with the previously observed slowdown in strength improvement at high fiber dosages.

### 3.4. Carbonate Production and Spatial Distribution Characteristics

**Figure 13:**
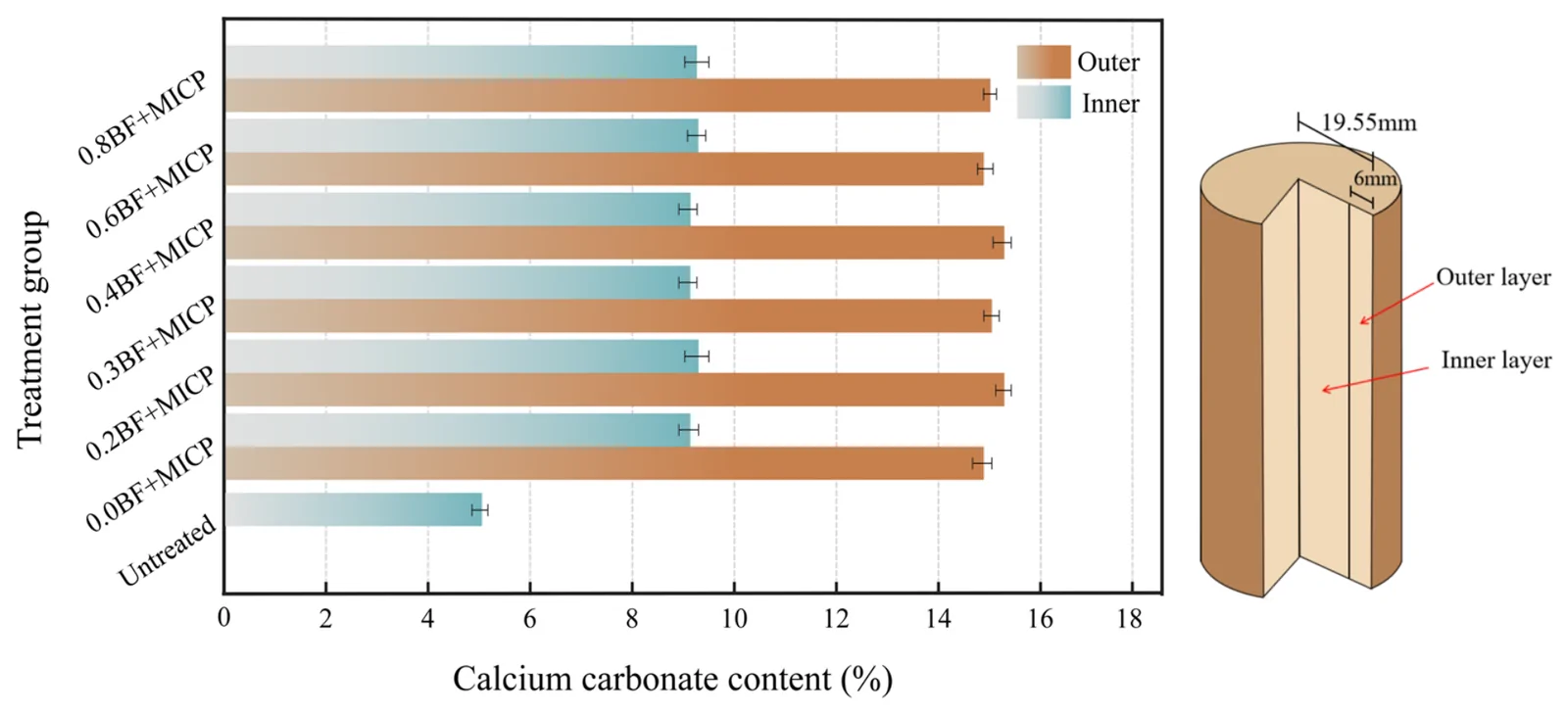
Spatial distribution of calcium carbonate (CaCO_3_) in BF-MICP-treated tailings at different basalt fiber contents. The outer region represents the approximately 6 mm-thick peripheral layer, whereas the inner region represents the remaining specimen core. Error bars represent the standard deviation of three parallel specimens.

In microbially based soil remediation, the amount of CaCO_3_ produced is positively correlated with soil strength [49,50]. Figure 13 shows the mass fraction of CaCO_3_ in specimens before and after BF-MICP treatment at different basalt fiber dosages, together with its spatial distribution between the outer layer and the inner region. Overall, MICP treatment increased the CaCO_3_ content in the tailings by approximately 2–3 times relative to the untreated group, whereas the total CaCO_3_ contents differed only slightly among different fiber dosages. Meanwhile, CaCO_3_ exhibited a pronounced “higher-outer/lower-inner” distribution within the soil column. These results suggest that CaCO_3_ production is primarily governed by biomineralization conditions and seepage pathways. The role of fibers is more likely to lie in modifying the deposition morphology and spatial structure rather than substantially increasing the total amount produced.

Compared with the untreated tailings, the average CaCO_3_ content of the BF-MICP-treated specimens increased from 4.8% to 13.9–14.0%. The treated groups had significantly higher CaCO_3_ contents than the untreated group (*p* < 0.001). However, no significant differences were found among the BF-MICP groups with different fiber dosages (*p* > 0.05), indicating that basalt fiber had no pronounced effect on the total CaCO_3_ yield. Therefore, the strength differences among groups are more likely attributable to differences in the spatial distribution and interconnection structure of fibers and CaCO_3_, rather than changes in the total amount of CaCO_3_ produced.

From a spatial perspective, the CaCO_3_ content in the outer layer was consistently higher than that in the inner region. Specifically, the outer-layer CaCO_3_ content remained at 15.1–15.3%, which was distinctly higher than the inner-layer value of 9.5–9.7%. This suggests that during cyclic grouting, the cementation solution flowed from the exterior toward the interior, and intensive MICP reactions first occurred in pores near the outer surface. CaCO_3_ precipitated rapidly and gradually caused pore clogging. The resulting clogging at the periphery increased the seepage resistance of the outer layer, thereby limiting the subsequent inward migration of the cementation solution and leading to relatively insufficient carbonate precipitation in the interior.

Although preferential cementation occurred near the specimen surface, the inner region also contained 9.5–9.7% CaCO_3_, approximately twice the value of the untreated tailings, and viable bacteria remained detectable in this region. Moreover, the outer region accounted for only approximately one-quarter of the total carbonate mass, indicating that the inner matrix was not mechanically or chemically negligible. Considering the CaCO_3_ content together with UCS, the increased load-bearing capacity under BF-MICP can be attributed to the cemented framework formed by CaCO_3_ between particles, while the fibers provided bridging and pull-out resistance and restrained crack propagation. Nevertheless, the individual mechanical contributions of the outer and inner regions were not separately quantified and require further investigation using layer-specific testing or X-ray computed tomography.

In conjunction with the mechanical results, although the total CaCO_3_ content changed little among different fiber dosages, UCS, peak strain, as well as residual stress and energy dissipation varied markedly with fiber dosage. This contrast indicates that the role of basalt fiber in BF-MICP is mainly reflected in regulating the spatial arrangement and cementation morphology of CaCO_3_: Fibers provided nucleation sites and attachment surfaces for CaCO_3_ precipitation, facilitating carbonate deposition at particle contact points and at the fiber–soil interface, and promoting a bridging-type cementation. As a result, a more continuous load-bearing framework was progressively established among tailings particles, CaCO_3_, and fibers. In addition, the enrichment of CaCO_3_ in the surface layer can form a relatively dense coating, reducing effective seepage pathways and structurally favoring an overall decrease in leached concentrations.

### 3.5. Microstructural Composition and Structural Analysis

#### 3.5.1. Mineral Phase Evolution

**Figure 14:**
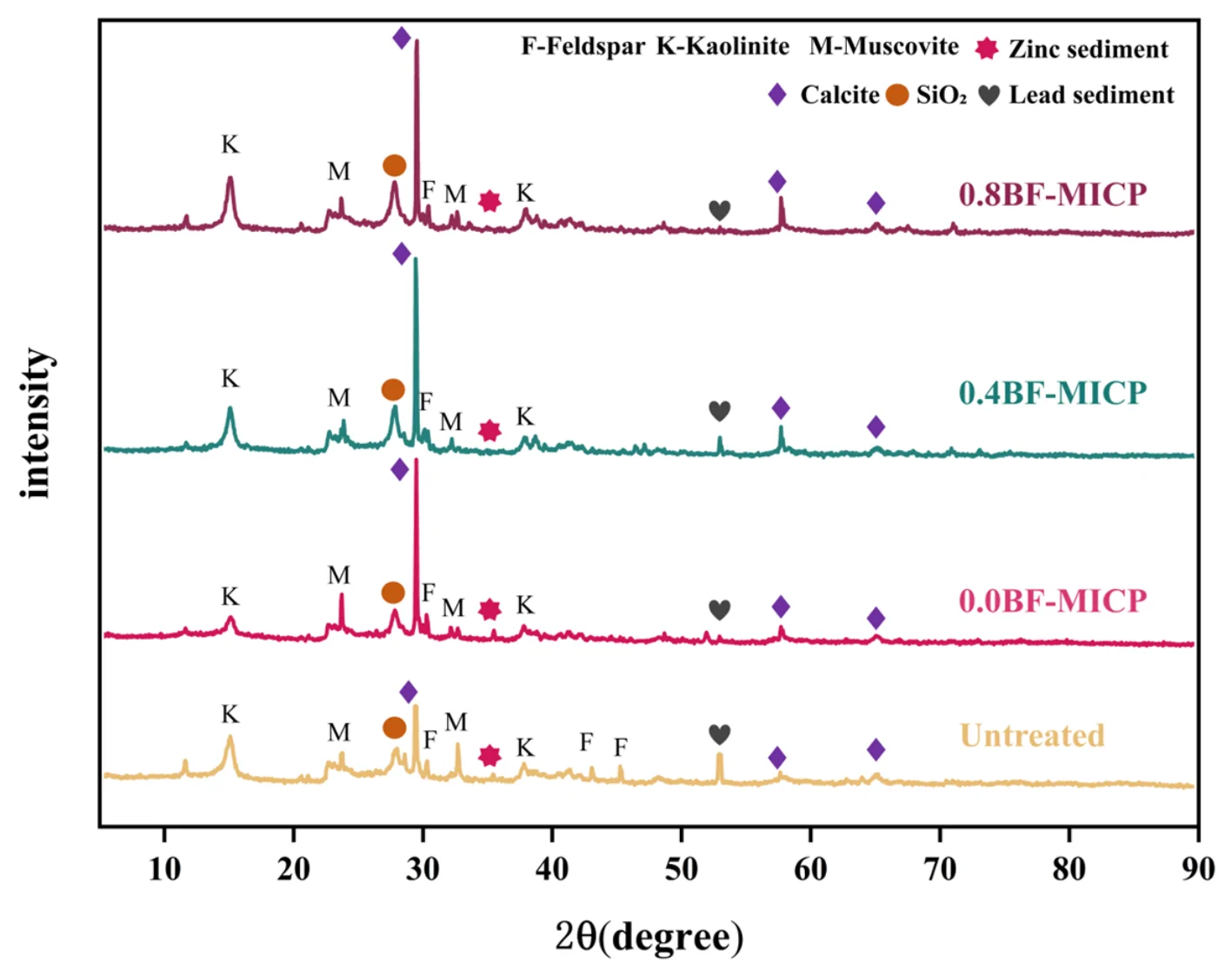
XRD patterns of tailings under different treatment conditions.

Figure 14 shows the XRD patterns of rare earth tailings under different treatment conditions. Mineral phases were identified based on the ICDD PDF database. All samples retained the characteristic reference peaks of the parent-rock minerals, including quartz (PDF#39-1910) at 26.6°, muscovite (PDF#07-0025) at 23.8°, kaolinite (PDF#14-0164) at 37.7–38.8°, and K-feldspar (PDF#19-0932) at 30.1° and 43.1°. These phases were generally consistent with those of the raw tailings. Only a slight decrease in the relative intensity of some peaks was observed in the treated groups, suggesting that the mineralization products mainly coated particle surfaces and produced a dilution effect, while the primary minerals themselves remained essentially unchanged.

After MICP treatment, all treated groups exhibited pronounced carbonate diffraction signals. Calcite showed a main peak at 29.4° [51], with accompanying peaks at 39.4°, 57.5°, and around 65°. The fiber-containing groups showed some differences in peak intensity and peak shape near 29.4°. These differences may be related to the crystallinity of calcite and can also be influenced by sample grinding and packing conditions. Considering the acid-leaching mass-loss results showing little difference in total CaCO_3_ among groups, these variations are more likely to reflect changes in deposition morphology and structural features rather than a substantial change in the total amount of CaCO_3_ produced.

Regarding heavy-metal immobilization, weak reflections associated with metal carbonates were observed near 36° and 53°. This suggests that Zn and Pb may be encapsulated by carbonate precipitates during precipitation, and a fraction may be incorporated into the carbonate crystal lattice, thereby transforming into less soluble forms. These weak reflections can overlap with peaks of feldspar and clay minerals. In addition, the signals are weak and the corresponding contents are low, making it difficult to reliably assign them to specific mineral phases. Therefore, no specific PDF card numbers were assigned to these reflections; they were only used as qualitative evidence that heavy metals participated in carbonate precipitation. The stabilization effect was further supported by the enrichment of Pb and Zn revealed by SEM-EDS and by the pronounced decreases in leached concentrations. Overall, BF-MICP treatment did not change the types of parent-rock minerals in the tailings. Instead, it induced the formation of a biomineralized cementation phase dominated by calcite with a higher degree of crystallinity, together with a small amount of metal carbonates and interfacial interactions involving clays and extracellular polymeric substances (EPS), thereby enhancing the immobilization of Pb^2+^ and Zn^2+^. This is consistent with the observed macroscopic mechanical enhancement and the reduction in leached concentrations.

#### 3.5.2. Functional Group Variations

**Figure 15:**
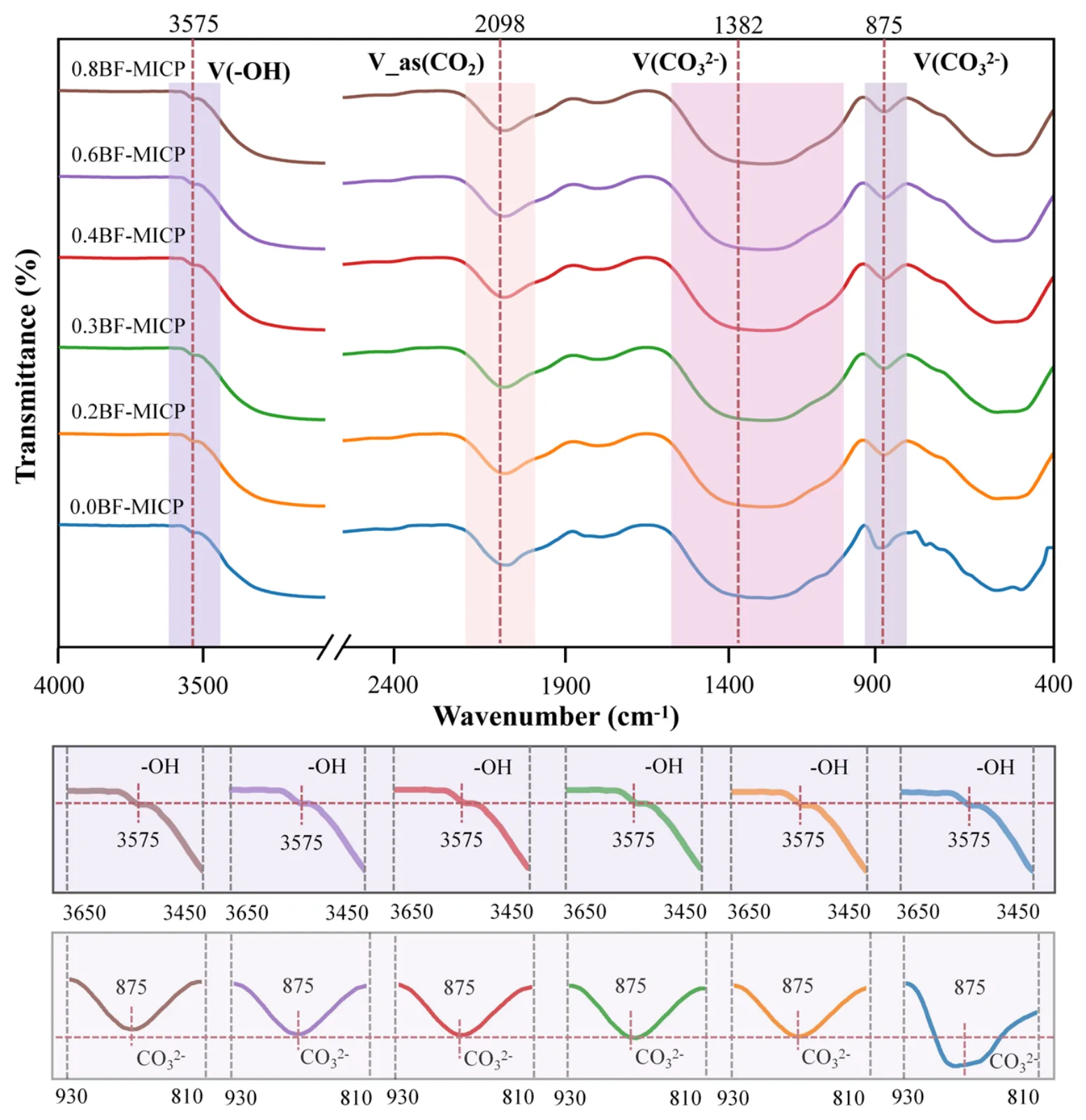
FTIR spectra of tailings under different treatment conditions.

Figure 15 shows the FTIR spectra of rare earth tailings under different treatment conditions. All treated groups exhibited a broad -OH stretching band near 3400 cm^−1^ and an H-O-H bending band of adsorbed water around 1600 cm^−1^. In the high-wavenumber region, a distinct -OH vibration band was also observed at 3575 cm^−1^, indicating the widespread presence of pore water and hydroxyl groups on clay surfaces.

After MICP and BF-MICP treatment, characteristic carbonate bands appeared clearly at 1382 cm^−1^ (CO_3_^2−^ stretching) and 875 cm^−1^ (CO_3_^2−^ out-of-plane bending) [52]. Their intensities increased markedly compared with the untreated group, indicating substantial CaCO_3_ formation within the specimens and on particle surfaces. As the basalt fiber dosage increased from 0.2% to 0.4%, the bands at 1382 and 875 cm^−1^ increased continuously in intensity and became slightly sharper, with the 0.4 BF group showing the most pronounced change. When the fiber dosage was further increased to 0.8%, the carbonate-band intensity decreased slightly. This suggests that an appropriate fiber content is more favorable for forming a continuous and dense CaCO_3_ deposition structure; in contrast, excessive fiber may promote agglomeration and locally increase porosity, which can restrict the effective extent of biomineral deposition and weaken the overall deposition effect.

Meanwhile, in the treated groups, the broad -OH band near 3400 cm^−1^ became slightly narrower and decreased in intensity, and the H-O-H bending band at 1600 cm^−1^ weakened accordingly. This suggests that part of the free water and weakly adsorbed water was reduced as the newly formed CaCO_3_ cementation system developed, leading to a denser pore structure and a thinner water film.

No new absorption bands attributable to organic functional groups were observed in the FTIR spectra. In addition, the spectral response of basalt fiber overlapped with that of the matrix minerals within the relevant wavenumber ranges. These observations indicate that introducing fibers did not lead to the formation of new functional groups; instead, the fibers mainly provided attachment interfaces for CaCO_3_ and helped optimize the cementation structure, rather than generating new reaction products through chemical reactions.

#### 3.5.3. Micro-Morphology

**Figure 16:**
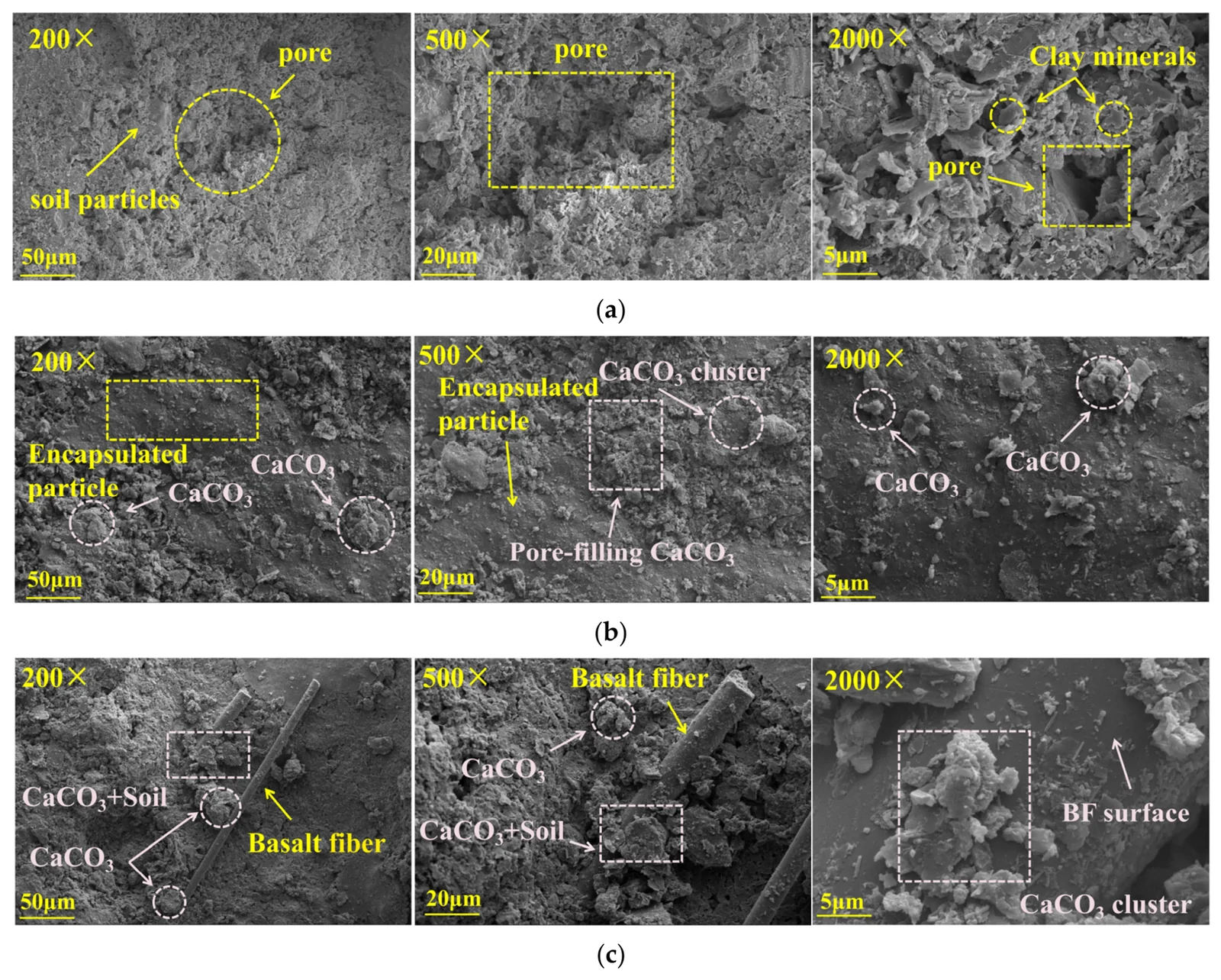
Representative SEM microstructures of tailings under different treatment conditions: (**a**) SEM microstructure of untreated tailings; (**b**) SEM microstructure of tailings after MICP treatment; (**c**) SEM microstructure of the mineralized interface in BF-MICP-treated tailings.

Figure 16 illustrates the microstructural evolution of rare earth tailings under different treatments, including the untreated condition, MICP solidification, and combined solidification using MICP with basalt fiber (BF). The SEM images were taken at magnifications of 200×, 500×, and 2000×, respectively. The images indicate that, with biomineralization and fiber incorporation, interparticle cementation was enhanced and the pore structure became denser.

At 200× and 500×, the untreated tailings consisted mainly of platy and granular aluminosilicate particles in a loose packing arrangement. Interparticle contacts were predominantly point-to-point with a small contact area, and particle boundaries were distinct. Numerous connected pore throats and microcracks were observed, suggesting relatively high pore connectivity. At 2000×, pore edges appeared sharp, particle surfaces were rough, and no obvious cementing products were observed. These features indicate a lack of effective cementation and continuous interparticle linkage, resulting in discontinuous stress transfer and a higher susceptibility to brittle failure.

For the MICP-solidified specimen, the structure appeared denser at low magnification. At 500×, discretely distributed CaCO_3_ precipitates were observed, mainly in the form of quasi-spherical crystal aggregates. A coating layer formed on the surfaces of some fine particles, and bridging-type cementation was also observed at pore-throat locations. At 2000×, CaCO_3_ deposition preferentially occurred at particle contact points, particle edges, and surface asperities/defects, forming localized cementation points. Although connectivity at these locations was enhanced to some extent, the deposits were not yet continuous, and the pores were not fully filled. This suggests that biomineralization remained at an early stage and the overall cemented network had not been fully established.

With the incorporation of BF, the overall microstructure was markedly improved. At 500×, CaCO_3_ precipitation was concentrated on fiber surfaces and at fiber ends, locally forming shell-like deposits accompanied by clustered aggregates. At 2000×, CaCO_3_ was preferentially deposited in the fiber–particle contact regions and formed bridging structures between them. CaCO_3_ cementation spanning pore spaces was also observed at some pore locations. More evident band-like deposits were observed along the fiber direction, suggesting that fibers provided attachment sites and guided the local growth of CaCO_3_, resulting in more continuous deposition and a larger coverage area. Biomineral deposits at pore boundaries, together with those on particle surfaces, may enhance interfacial bonding and structural stability. This, in turn, can promote the immobilization of Zn^2+^ and Pb^2+^ and reduce their mobility.

In the untreated tailings, the Ca elemental mapping signal was weak and scattered, whereas Zn and Pb signals were relatively uniform and dense, indicating limited Ca sources and a widespread distribution of heavy metals on particle surfaces without effective immobilization. After BF-MICP treatment, the Ca distribution was markedly enhanced with a network-like spread, while Zn and Pb signals decreased noticeably and became more localized, suggesting that microbially induced CaCO_3_ precipitation formed extensively and coated particle surfaces, thereby substantially reducing the mobility of heavy-metal ions.

**Figure 17:**
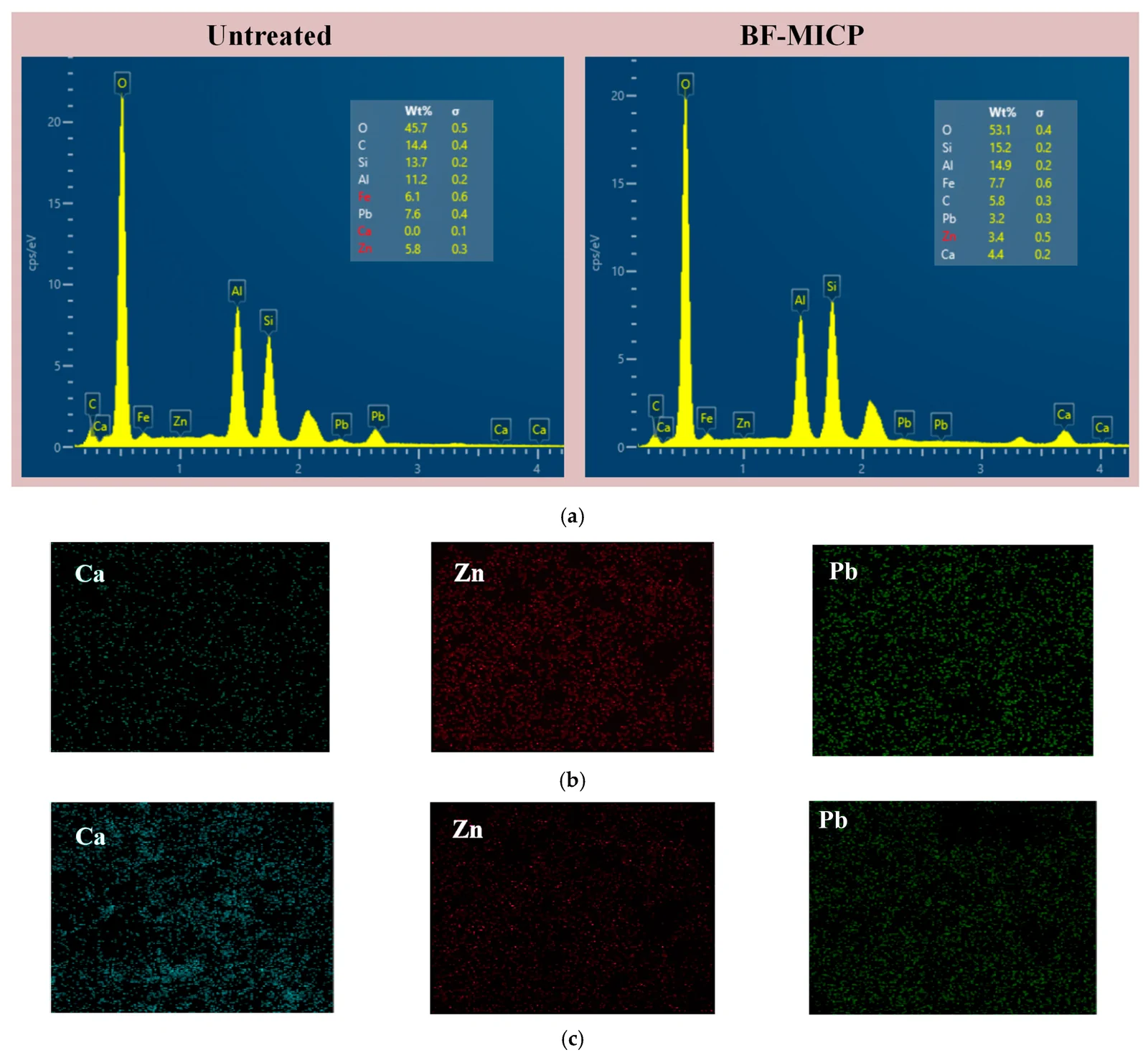
EDS spectra and elemental distribution maps of rare earth tailings before and after BF-MICP treatment. (**a**) Comparison of EDS spectra of tailings before and after BF-MICP treatment. (**b**) Spatial distribution of Ca, Pb and Zn in untreated tailings. (**c**) Spatial distribution of Ca, Pb and Zn in BF-MICP treated tailings.

The EDS spectra in Figure 17a further support this trend: the Untreated sample shows pronounced Zn and Pb peaks with a comparatively weak Ca peak, whereas the BF-MICP sample exhibits a much stronger Ca peak and weakened Pb and Zn peaks that approach the background level. The elemental maps in Figure 17 b-c are consistent with these observations. These results indicate more evident Ca enrichment and relatively weakened Pb and Zn signals after BF-MICP treatment. The underlying mechanisms may include adsorption and/or encapsulation of Pb^2+^ and Zn^2+^ by bacterially induced CaCO_3_ deposits, co-precipitation into more stable phases, and possible binding of metal ions by EPS, which together contribute to metal retention and isolation.

### 3.6. Solidification/Stabilization Mechanisms

**Figure 18:**
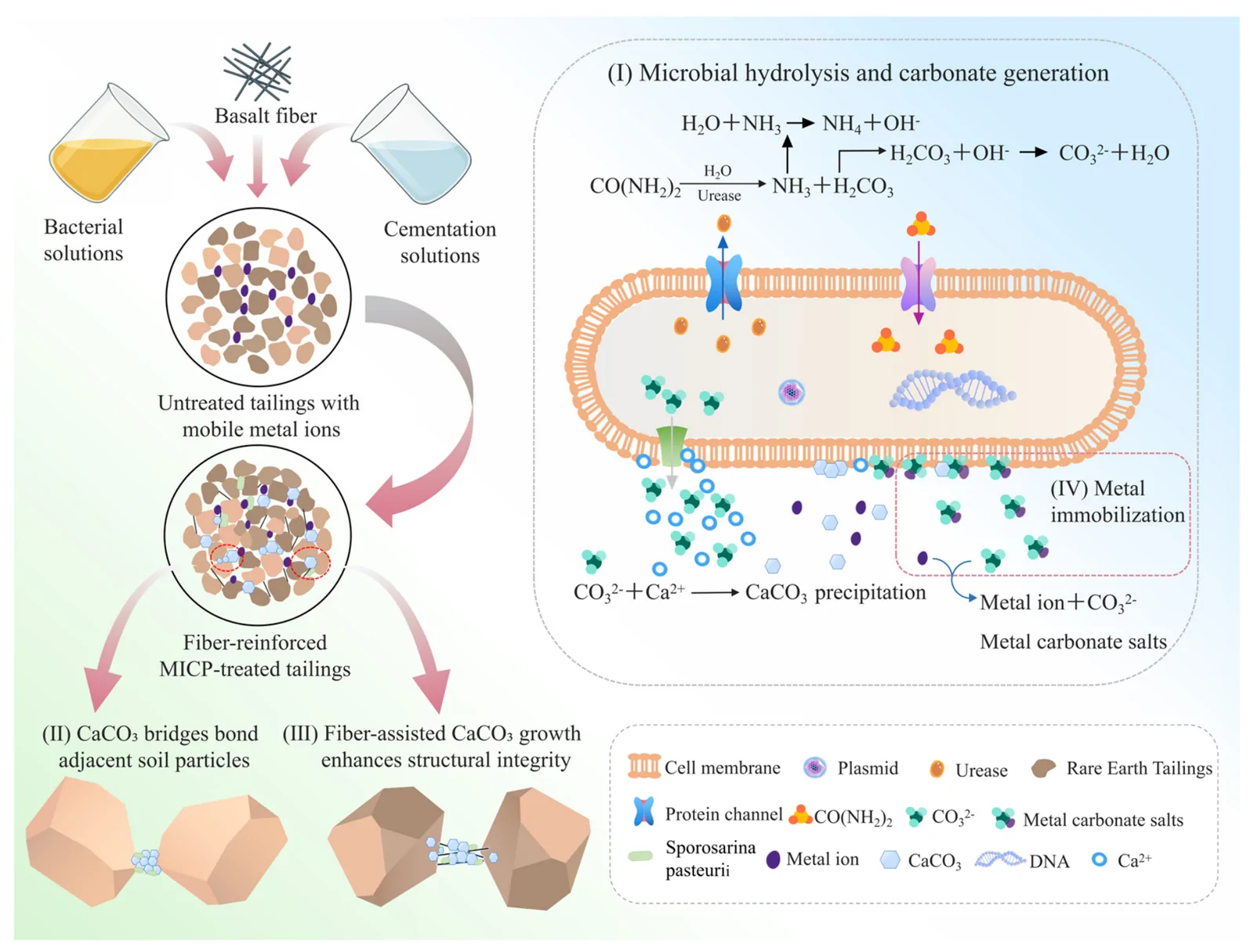
Schematic of the solidification/stabilization mechanisms of BF-MICP-treated ion-type rare earth tailings: (I) microbial hydrolysis and carbonate generation; (II) CaCO_3_ bridging between adjacent particles; (III) fiber-assisted CaCO_3_ growth and structural reinforcement; and (IV) Pb and Zn immobilization. BF-MICP, basalt-fiber-reinforced microbially induced carbonate precipitation.

The schematic illustration of the solidification and stabilization mechanisms of BF-MICP for ion-type rare earth tailings is shown in Figure 18. It can be summarized into four key processes: (1) microbial reactions supply carbonate ions; (2) CaCO_3_ precipitates within pores and cements particles, promoting densification; (3) basalt fibers participate in load transfer and restrain crack propagation; and (4) Pb^2+^ and Zn^2+^ are adsorbed and/or encapsulated during carbonate deposition, resulting in reduced mobility. The results of the sterile-medium and killed-cell controls are summarized in Table 7 to distinguish biologically mediated MICP from the physical and abiotic effects associated with fluid treatment.

As shown in Table 7, the 0.4 BF-SM group exhibited a UCS of 0.76 MPa and a CaCO_3_ content of 5.33%, while the Pb and Zn TCLP concentrations were 16.97 and 18.03 mg/L, respectively. Compared with the untreated tailings, the Pb and Zn leaching concentrations decreased by 24.9% and 18.4%, respectively. The 0.4 BF-KB group exhibited a UCS of 0.93 MPa and a CaCO_3_ content of 8.06%, while the Pb and Zn TCLP concentrations decreased to 11.31 and 11.28 mg/L, corresponding to reductions of approximately 50.0% and 49.0%, respectively.

The intermediate responses of the sterile-medium and killed-cell controls indicate that fluid treatment, solution chemistry, inactive cellular materials, and possible abiotic carbonate precipitation contributed partly to strength development and heavy-metal retention. However, both control groups exhibited lower UCS and CaCO_3_ contents and higher Pb and Zn leaching concentrations than the live-bacteria 0.4 BF-MICP group. These results support that active ureolysis and microbially mediated carbonate precipitation were the dominant contributors to the overall solidification/stabilization performance, whereas the non-biological contributions were secondary.

Stage I (microbial hydrolysis and carbonate generation). The bacterial suspension and cementation solution infiltrate the pore space of the tailings. Urease-producing bacteria adhere to tailings particle surfaces and the outer walls of basalt fibers, while extracellular polymeric substances (EPS) and negatively charged cell membranes provide enrichment sites for Ca^2+^. During curing, bacterial urease catalyzes urea hydrolysis to generate NH^4+^ and CO_3_^2−^. The hydrolysis raises pore-water pH and drives dissolved inorganic carbon toward CO_3_^2−^, leading to a rapid increase in CO_3_^2−^ concentration. As illustrated in region (I) of Figure 18, carbonate precipitation preferentially initiates on bacterial surfaces, fiber surfaces, and rough areas of tailings particles when CO_3_^2−^ encounters Ca^2+^ in solution. The initial precipitates are typically fine-grained with relatively imperfect crystals, providing a basis for subsequent CaCO_3_ growth and cementation.

Stage II (CaCO_3_ precipitation and particle cementation). The early, fine carbonate deposits continue to grow and gradually evolve into more stable crystals dominated by calcite. The concurrent enhancement of the main calcite diffraction peak in XRD and the carbonate vibration bands at 1382 and 875 cm^−1^ in FTIR reflects this evolution in crystal form and the increase in carbonate content. Newly formed CaCO_3_ deposits at particle contacts and pore walls, commonly as coating layers or localized bridging cementation. With continued deposition, fine crystals coarsen and interconnect, forming a more continuous cemented layer between particles and transforming the initially loose packing into an integrated structure with stronger interparticle linkage. Meanwhile, pore channels are partially filled, pore connectivity decreases, and the structure becomes denser. As shown in region (II) of Figure 18, CaCO_3_ first forms bridges at particle contacts, followed by outward growth that progressively strengthens and connects the interparticle cementation.

Stage III (fiber-involved structural reinforcement). Short-cut basalt fibers, as a high-strength aluminosilicate inorganic material, have a rough surface with hydrophilic groups such as hydroxyls. This favors bacterial attachment and Ca^2+^ enrichment, and also facilitates continuous CaCO_3_ deposition along the fiber direction. The experimental results indicate that at fiber dosages of 0.3–0.4%, CaCO_3_ tends to deposit more continuously on fiber surfaces and connect with the biomineral products on particle surfaces. A relatively continuous linkage network is thus formed among fibers, CaCO_3_, and tailings particles, which is conducive to improving load-bearing capacity and crack resistance. When the fiber dosage is excessive, fibers are more prone to agglomeration and local pore accumulation, weakening this linkage network and thereby reducing the overall effectiveness. As depicted in region (III) of Figure 18, CaCO_3_ distributed along basalt fibers connects adjacent particles, confining initially loose particles within the same supporting system.

Stage IV (synergistic heavy-metal immobilization). Pb^2+^ and Zn^2+^ can be immobilized through multiple pathways in an alkaline carbonate environment. On the one hand, Pb^2+^ and Zn^2+^ may react with CO_3_^2−^ to form sparingly soluble carbonate precipitates, and a fraction may be incorporated into the calcite lattice, leading to reduced mobility. On the other hand, EPS, residual biomass, and CaCO_3_ deposition collectively reduce pore channels and densify the structure, allowing heavy metals to be immobilized within the cemented framework via adsorption and/or encapsulation. At the macroscale, this is manifested by markedly reduced Pb and Zn leached concentrations that remain below the leaching limits.

The mechanisms of BF-MICP for treating ion-type rare earth tailings can be summarized as follows. First, urease-producing bacteria continuously supply CO_3_^2−^ at particle surfaces and interfaces and induce CaCO_3_ deposition predominantly in the form of calcite, thereby strengthening inorganic cementation between particles. Second, basalt fibers provide attachment sites for CaCO_3_ precipitation, allowing dispersed deposits to connect more readily with surrounding particles; consequently, interparticle bonding becomes stronger, cracks are less likely to rapidly coalesce, and brittle failure is alleviated. Third, within this cemented framework, Pb^2+^ and Zn^2+^ can be adsorbed and/or encapsulated during carbonate deposition, resulting in reduced mobility. Collectively, these processes lead to clear improvements in strength, deformation capacity, and TCLP leaching safety compared with the untreated state.

From an engineering perspective, the service performance of BF-MICP-treated tailings is expected to depend primarily on the persistence of CaCO_3_ cementation and fiber–matrix bonding under acidic infiltration, wet–dry cycling, and sustained or cyclic loading. Potential long-term deterioration mechanisms include carbonate dissolution, progressive interfacial damage, redistribution of Pb and Zn after mineral dissolution, and the release of ammonium generated during urea hydrolysis. Because the present study was based on short-term laboratory treatment and leaching evaluation, a quantitative design service life cannot yet be assigned.

For field application, quality control should combine initial acceptance testing with periodic performance monitoring. Recommended indicators include UCS or penetration resistance, hydraulic conductivity, pore-water pH, CaCO_3_ content, ammonium release, and Pb/Zn leaching concentrations. Monitoring frequency and acceptance thresholds should be established according to the site exposure conditions, while long-term wet–dry cycling, acidic leaching, and repeated-loading tests are required before large-scale engineering application.

## 4. Conclusions

(1) BF-MICP markedly improved the unconfined compressive performance of ion-type rare earth tailings. Both UCS and peak strain exhibited an “increase-decrease” trend with increasing basalt fiber dosage. At fiber dosages of 0.3–0.4%, the specimens showed relatively high peak strength and residual stress, and the failure mode shifted from a single through-going splitting crack to multi-crack shear-slip behavior, accompanied by enhanced ductility and energy dissipation capacity.
(2) BF-MICP exhibited a significant stabilization effect on Pb and Zn. Compared with the untreated tailings, the leaching concentrations of Pb and Zn decreased by approximately 80% after both MICP-only and BF-MICP treatment, and all values were below the leaching limits. Meanwhile, Pb and Zn were transformed from acid-extractable fractions into more stable residual fractions. Only minor differences were observed among the BF-MICP groups with different fiber contents, indicating that basalt fiber did not further reduce the leaching levels compared with MICP alone.
(3) Considering both the macroscopic mechanical performance and the Pb/Zn TCLP results, a basalt fiber dosage of 0.3–0.4% is recommended as an optimal range for BF-MICP treatment of ion-type rare earth tailings to balance load-bearing performance and environmental safety.
(4) CaCO_3_ produced by MICP provided the primary material basis for tailings solidification/stabilization, whereas fibers mainly regulated its spatial distribution. Relative to the untreated tailings, the CaCO_3_ mass fraction increased by approximately 2–3 times after treatment and exhibited an outer-high/inner-low gradient. Basalt fibers supplied attachment sites for CaCO_3_, promoting a more developed cemented linkage between fibers and particles. The sterile-medium and killed-cell controls produced only partial improvements in strength, carbonate production, and heavy-metal retention and did not reproduce the performance of the live-bacteria BF-MICP treatment, further supporting the dominant role of active ureolysis.
(5) Microstructural characterization indicates that BF-MICP treatment was dominated by calcite-type CaCO_3_ precipitation, and that CaCO_3_ together with basalt fibers strengthened interparticle connectivity and improved structural densification. SEM-EDS further showed that Pb^2+^ and Zn^2+^ were mainly associated with biomineralized deposition zones and regions near the fiber interface, suggesting a greater tendency for heavy metals to be retained and immobilized.

## Figures and Tables

**Table 1:** Physical properties of the tested tailings.

Water Content ω%	Density ρ/(g/cm^3^)	Liquid Limit W_L_ (%)	Plastic Limit W_P_ (%)	pH Value	Plasticity Index I_P_
25.73	1.72	41.83	29.23	5.59	12.60

**Table 2:** Major chemical composition of the rare earth tailings.

Composition	Na_2_O	MgO	Al_2_O_3_	SiO_2_	P_2_O_5_	K_2_O	TiO_2_	Fe_2_O_3_	ZnO	PbO
Content (%)	0.14	0.58	32.85	39.82	0.26	1.85	1.56	4.33	0.17	0.19

**Table 3:** Physical properties of basalt fibers.

Length mm	Diameter μm	Density g/cm^3^	Elastic Modulus GPa	Tensile Strength MPa	Elongation at Break %
6	18	2.60–2.80	≥90	>1050	<3.5

**Table 4:** Composition of the cementation solution.

CO(NH_2_)_2_ (g)	CaCl_2_ (g)	NH_4_Cl (g)	NaHCO_3_ (g)	Nutrient Broth (g)	PH	Deionized Water (mL)
60.0	113.0	10.0	2.0	3.0	7.2	1000

**Table 5:** Urease activity and viable bacterial abundance in tailings during treatment and curing.

Time	Relative Urease Activity (%)	Outer Viable Bacteria [log_10_(CFU·g^−1^)]	Inner Viable Bacteria [log_10_(CFU·g^−1^)]	Outer/Inner Ratio	Background Urease Activity in Non-Inoculated Tailings (%)
Day 0	100.0	7.94	7.89	1.12	3.1
Day 7	44.7	6.71	6.49	1.66	16.2
Day 14	18.9	5.79	5.68	1.29	13.8

**Table 6:** Specimen groups and treatment conditions.

Samples	Fiber Content (%)	Bacterial Component	Cementation Solution (Urea and CaCl_2_) (mol/L)
Untreated	0	None	0
0.0 BF-MICP	0	Active bacterial suspension	1
0.2 BF-MICP	0.2	Active bacterial suspension	1
0.3 BF-MICP	0.3	Active bacterial suspension	1
0.4 BF-MICP	0.4	Active bacterial suspension	1
0.6 BF-MICP	0.6	Active bacterial suspension	1
0.8 BF-MICP	0.8	Active bacterial suspension	1
0.8 BF-only	0.8	None	0
0.4 BF-SM	0.4	Sterile medium	1
0.4 BF-KB	0.4	Killed-cell suspension	1

Note: SM, sterile-medium control; KB, killed-cell control. The active and killed-cell suspensions were prepared at the same initial OD_600_ of 0.98.

**Table 7:** Comparison of treatment effects at 0.4% basalt fiber content.

Group	UCS (MPa)	CaCO_3_ Content (%)	Pb TCLP (mg/L)	Zn TCLP (mg/L)
Untreated	0.58 ± 0.02	4.80 ± 0.16	22.60 ± 0.70	22.10 ± 0.84
0.4 BF-SM	0.76 ± 0.04	5.33 ± 0.10	16.97 ± 0.37	18.03 ± 0.30
0.4 BF-KB	0.93 ± 0.03	8.06 ± 0.32	11.31 ± 0.45	11.28 ± 0.43
0.4 BF-MICP	1.72 ± 0.07	14.00 ± 0.23	4.70 ± 0.13	4.40 ± 0.08

## Data Availability

The original contributions presented in this study are included in the article. Further inquiries can be directed to the corresponding author.
